# Self‐Care Behaviors in Osteoporosis: A Cross‐Cultural Study Using Multidimensional Analysis of Textual Data

**DOI:** 10.1111/jnu.70134

**Published:** 2026-09-14

**Authors:** Chiara Tedesco, Vicente Bernalte‐Martí, Adela Melgarejo Uréndez, Arantxa Conesa, María Desamparados Bernat‐Adell, Mara Tormen, Gianluca Pucciarelli, Ercole Vellone, Maddalena De Maria, Emanuela Basilici Zannetti, Noemi Cittadini, Annalisa Pennini, Umberto Tarantino, Rosaria Alvaro, Mariachiara Figura

**Affiliations:** ^1^ Department of Biomedicine and Prevention University of Rome Tor Vergata Roma Italy; ^2^ Department of Nursing, Faculty of Health Sciences University of Jaume I Castellón Spain; ^3^ Documentación Clínica y Admisión Unit Hospital General Universitario de Castellón Castellón Spain; ^4^ Rheumatology Department Hospital General Universitario de Castellón Castellón Spain; ^5^ Medicine Department, Faculty of Health Sciences University of Jaume I Castellón Spain; ^6^ Division of Research Methodology, Department of Nursing, Faculty of Nursing and Midwifery Wroclaw Medical University Wrocław Poland; ^7^ Department of Life Health Sciences and Health Professions Link Campus University Rome Italy; ^8^ Department of Orthopaedics and Traumatology Policlinico Tor Vergata Foundation University of Rome Tor Vergata Roma Italy; ^9^ University “Our Lady of Good Counsel” Tirana Albania; ^10^ Department of Maternal and Child Health Promotion, Internal Medicine & Specialties of Excellence “G. D'Alessandro” (PROMISE) University of Palermo Palermo Italy

**Keywords:** cross‐cultural, multidimensional analysis, osteoporosis, qualitative, self‐care

## Abstract

**Introduction:**

Osteoporosis is a chronic skeletal condition that significantly affects daily life and may benefit from adequate self‐care. The Middle‐Range Theory of Self‐Care of Chronic Illness conceptualizes self‐care as a naturalistic process encompassing maintenance, monitoring, and management, also shaped by contextual and cultural factors. Despite this theoretical framework, limited evidence exists on how people with osteoporosis conceptualize and enact self‐care in different cultural contexts. Italy and Spain provide comparable yet distinct settings for exploring cultural influences on self‐care practices. This study aims to explore and compare self‐care behaviors among Italian and Spanish people with osteoporosis.

**Design:**

A cross‐national qualitative study was conducted using an Automatic Analysis of Textual Data approach within the Exploratory Multidimensional Data Analysis framework.

**Methods:**

Forty people with osteoporosis (38 women and 2 men; mean age 68.6 years, SD = 5.74) were recruited, equally distributed between Italy (*n* = 20) and Spain (*n* = 20). Data were analyzed using Labbé's intertextual distance and correspondence analysis to examine lexical similarities, differences, and semantic structures of self‐care narratives.

**Results:**

Although moderate intra‐national lexical similarity was observed within each national group, substantial inter‐national differences emerged between Italian and Spanish participants in the discursive construction of self‐care. Italian participants described self‐care as a medically mediated and system‐oriented process, closely linked to professional guidance, diagnostic procedures, and prescribed treatments. In contrast, Spanish participants framed self‐care as embodied, routine‐based, and emotionally expressive, grounded in daily practices and bodily awareness. These findings indicate that participants relied on distinct cultural and epistemic models when interpreting and organizing self‐care experiences.

**Conclusion:**

This study highlights clear cross‐cultural differences in how people with osteoporosis narrate and conceptualize self‐care. Methodologically, it illustrates the potential of multidimensional analysis to support systematic and transparent comparison of qualitative datasets. The findings are consistent with the Middle‐Range Theory of Self‐Care and suggest that greater attention to cultural contexts may inform the development of self‐care interventions that are more responsive to individuals' lived experiences.

**Clinical Relevance:**

Recognizing how cultural contexts influence how people with osteoporosis understand and practise self‐care may help nurses tailor communication and educational support to individuals' everyday experiences, supporting more responsive and culturally sensitive care.

**Abbreviations**
AATD: Automatic Analysis of Textual Data
CA: correspondence analysis
EMDA: Exploratory Multidimensional Data Analysis
PwOP: people with osteoporosis
TTR: Type–Token Ratio

## 1 Introduction

Osteoporosis is a chronic and progressive skeletal condition characterized by reduced bone mass and deterioration of bone microarchitecture, which increases bone fragility and susceptibility to fractures. Affecting more than 200 million individuals worldwide, its prevalence is steadily rising due to population aging, making it a major clinical and public health challenge (Hemmati et al. 2021; Kanis et al. 2021).

Osteoporosis is predominantly a condition of older age and of women. The probability of low‐trauma fracture increases with age in both sexes, though women bear a disproportionate burden: at Age 45, fracture risk reaches 47.3% in women compared to 23.8% in men in Western Europe, and in women this risk exceeds that of breast cancer (Tarantino et al. 2017). This heightened vulnerability is largely driven by postmenopausal bone loss, resulting from the decline in estrogen that disrupts the balance between bone resorption and formation (Hemmati et al. 2021). Senile osteoporosis, affecting both sexes in later life, further compounds this burden. Indeed, aging itself represents a critical contextual factor: older adults typically face progressive functional decline, increased comorbidity burden, and greater dependence on healthcare systems, all of which shape the lived experience of chronic conditions such as osteoporosis (Johnston and Dagar 2020; Tarantino et al. 2017).

Fragility fractures, the most severe clinical manifestation of the disease, often lead to chronic pain, mobility limitations, functional decline, and greater functional dependence, profoundly affecting individuals' autonomy and overall quality of life (Johnston and Dagar 2020). The psychological impact is also substantial, as fear of falling, loss of confidence, and withdrawal from daily activities commonly emerge, shaping patients' perceptions of vulnerability and influencing their engagement in health‐related behaviors (Dziedzic et al. 2022).

Despite its considerable burden, osteoporosis is frequently described as a “silent disease,” as symptoms typically remain unnoticed until a fracture occurs. This lack of early warning signs contributes to delayed diagnosis and limited opportunities for timely intervention (Hemmati et al. 2021; Johnston and Dagar 2020). Once the condition is identified, people with osteoporosis (PwOP) should incorporate into their daily routine a variety of self‐directed health behaviors, including physical activity, dietary adjustments, medication adherence, and symptom monitoring, to prevent subsequent fractures and preserve functional autonomy (Arceo‐Mendoza and Camacho 2021; Basilici Zannetti et al. 2017; Subarajan et al. 2024; Tarantino et al. 2017; Tedesco, Bernalte‐Martí, Pucciarelli, et al. 2025). In this context, self‐care becomes a key component of disease management in the post‐diagnosis phase.

According to the Middle‐range Theory of Self‐Care of Chronic Illness (Riegel et al. 2012, 2026), self‐care is a naturalistic decision‐making process in disease management encompassing three dimensions: self‐care maintenance (i.e., enacting health‐promoting and preventive behaviors), self‐care monitoring (i.e., paying attention to psychophysical changes in own's own body), and self‐care management (responding appropriately to symptoms). Evidence from chronic illnesses consistently demonstrates that effective self‐care behaviors improve treatment adherence, enhance symptom control, reduce hospitalizations, and promote better quality of life (Caggianelli et al. 2022; Lee et al. 2022; Rebora et al. 2021; Riegel et al. 2017). This theory also emphasizes that self‐care is influenced by contextual factors, including cultural norms, social expectations, family roles, and healthcare system characteristics. Research on chronic conditions confirms that cultural environments influence how individuals interpret bodily sensations, attribute meaning to symptoms, engage with treatment recommendations, and navigate daily health practices (Osokpo and Riegel 2021; Zeng et al. 2023).

Despite these insights, little is known about how PwOP conceptualize and enact self‐care within their cultural context. To date, only a few previous studies have applied the Middle‐Range Theory of Self‐Care of Chronic Illness to osteoporosis (Bernalte‐Martí et al. 2026; Cittadini et al. 2021; Tedesco, Bernalte‐Martí, Pucciarelli, et al. 2025; Tedesco, Bernalte‐MartÍ, Tormen, et al. 2025; Tormen et al. 2025), and no research has examined these behaviors through a cross‐cultural approach. This represents a critical gap, particularly because the condition poses distinctive challenges for self‐care: its subtle and often imperceptible symptomatology requires individuals to interpret subtle psychophysical cues, whereas the constant risk of future fractures demands sustained vigilance and continuous behavioral adaptation. In such a context, cultural norms surrounding food, body awareness, physical activity, family support, and attitudes toward aging may substantially shape how individuals conceptualize, prioritize, and enact self‐care.

Italy and Spain offer two comparable yet culturally distinct contexts for exploring how cultural environments influence self‐care in PwOP. Although both countries share universal healthcare systems and similar epidemiological profiles, they differ in health behaviors, perceptions, and outcomes, shaped by varying attitudes, for instance toward food, end of life care, mental health, family roles, and responses to stress (Bersia et al. 2024; Giannotti et al. 2022; Meñaca et al. 2012; Scarpato et al. 2022). Moreover, studies conducted in Italy and Spain have examined self‐care behaviors, though primarily in conditions other than osteoporosis. In both countries, medication adherence and attendance at clinical appointments consistently emerged as the most performed self‐care maintenance behaviors, whereas physical activity was the least performed in Italian diabetes patients and Spanish heart failure patients (Ausili et al. 2017; Juárez‐Vela et al. 2021), and stress management the least performed in Italian patients with inflammatory bowel disease (Napolitano et al. 2025). Regarding self‐care monitoring, Italian patients with inflammatory bowel disease and cancer reported adequate symptom tracking, largely structured around scheduled clinical visits and diagnostic examinations (Di Nitto et al. 2022; Napolitano et al. 2025), whereas Spanish patients with multiple chronic conditions demonstrated notably high monitoring scores (Durán‐Gómez et al. 2023). Finally, regarding self‐care management behaviors, suboptimal scores were documented in Italian patients with inflammatory bowel disease and heart failure (Ausili et al. 2017; Napolitano et al. 2025; Vellone et al. 2020), and in Spanish heart failure patients and their caregivers, with contacting healthcare providers as the most performed behavior and autonomous symptom management as the least performed across both countries (Antonio‐Oriola et al. 2022; Durán‐Gómez et al. 2023; Juárez‐Vela et al. 2021). However, no study has directly compared self‐care behaviors between Italian and Spanish populations, and no evidence exists in the specific condition of osteoporosis. Understanding these culturally embedded differences is essential for informing educational and supportive interventions that are not only clinically appropriate but also culturally attuned.

To address these gaps, this study employs an innovative data analysis approach, grounded in Exploratory Multidimensional Data Analysis (EMDA), to explore and compare self‐care narratives among Italian and Spanish adults living with osteoporosis. By analyzing large qualitative datasets in a systematic and reproducible way, the study aims to generate the first cross‐cultural evidence on how PwOP understand, monitor, and manage their condition, offering new theoretical and clinical insights for tailored, culturally sensitive nursing interventions.

## 2 Design

A qualitative cross‐national study was conducted to explore self‐care behaviors among Italian and Spanish people living with osteoporosis. Semi‐structured interviews were developed according to the Middle‐Range Theory of Self‐Care of Chronic Illness, investigating the three domains of self‐care maintenance, self‐care monitoring, and self‐care management (Riegel et al. 2012, 2026).

This study adopts an interpretive perspective on textual data, viewing meaning as emerging from how discourse is organized across texts rather than from isolated individual accounts. Interviews are treated as a shared linguistic space in which experiences are expressed and shaped through language. The analysis focuses on recurring discursive patterns in the corpus (Fairclough 2013; Gee 2011; Reinert 1990; Ricoeur 1976). Quantitative textual patterns orient qualitative interpretation, with meaning developing through the interaction between these structured outputs and the researchers' interpretive judgment.

Data were analyzed using Automatic Analysis of Textual Data (AATD) within the broader framework of Exploratory Multidimensional Data Analysis (EMDA) (Bolasco 2021; Fraire 2009). This approach integrates multidimensional statistical analysis with qualitative interpretation, enabling researchers to explore how participants attribute meanings through the vocabulary they use (Figura et al. 2024). AATD is grounded in the EMDA framework, which aims to identify the underlying structure of discourse through the formalization of lexical co‐occurrences and their organization within a multidimensional space (Bolasco 2021; Lebart et al. 1998; Reinert 1990). Within this framework, textual data are not treated as isolated narratives but as components of a structured corpus, allowing the exploration of latent dimensions, oppositions, and relational patterns that characterize how meanings are constructed and distributed across the dataset. Within this perspective, EMDA is not only a set of analytical techniques, but a way to formally model discourse as a structured relational system. EMDA has been widely applied in health and social research to investigate how individuals and groups construct and negotiate meanings around complex phenomena, including health experiences, care practices, and professional roles (Figura et al. 2023, 2026; Konopka et al. 2018; Lo Monaco et al. 2026; Piervisani et al. 2025). In particular, its application through software such as IRaMuTeQ enables the integration of statistical modeling and interpretive analysis, supporting a systematic exploration of discourse that goes beyond purely descriptive qualitative approaches. In the context of the present study, EMDA was employed to examine how self‐care is discursively constructed among older adults across different national contexts, focusing on the relational organization of meanings rather than on the identification of isolated themes.

Within the EMDA framework, two complementary analytical techniques were applied. Labbé's intertextual distance was first used to examine both intra‐group lexical cohesion and inter‐group divergence between Italian and Spanish narratives, based on word frequency distributions (Cortelazzo et al. 2013; Labbé and Labbé 2003). Subsequently, correspondence analysis (CA) was used to map associations (chi‐square, *χ* ^2^) between words and categorical variables (e.g., nationality or self‐care dimensions), thereby identifying discursive patterns and latent semantic dimensions that structured participants' accounts. This approach enabled a systematic and reproducible cross‐national comparison, allowing for the identification of culturally embedded differences in self‐care narrative, ensuring that the analysis remained grounded in participants' actual language use while supporting theoretically informed interpretation (Figura et al. 2023). The EMDA framework has gained increasing relevance in nursing and social research (Figura et al. 2024, 2025; Piervisani et al. 2025; Veronese et al. 2025), and it is particularly suited to large and cross‐national datasets, where it helps uncover both shared meanings and culturally specific discourse patterns (Durante et al. 2022; Figura et al. 2024). This study further advances this line of research by integrating Labbé's distance and CA within the domains of the Middle‐Range Theory of Self‐Care of Chronic Illness, an application not previously reported in the nursing literature. To ensure methodological transparency and rigor, the study adhered to the Consolidated Criteria for Reporting Qualitative Research (COREQ) checklist (Tong et al. 2007).

## 3 Materials and Methods

### 3.1 Sampling and Recruitment

Participants were recruited in Italy and Spain from public hospital outpatient clinics specialized in osteoporosis management. Recruitment followed a purposive strategy to ensure variation in age, clinical history, and experiences of living with osteoporosis. Specifically, participants were selected to span a broad age range (55–85 years), consistent with the epidemiological profile of osteoporosis in Europe, where the condition predominantly affects adults aged 50–84 years (Hernlund et al. 2013), to represent different pharmacological treatments (including bisphosphonates, denosumab, teriparatide, and calcium and vitamin D supplementation), different years of diagnosis, different fracture histories (with and without fragility fractures, affecting different skeletal sites), different comorbidity profiles, and different everyday self‐care practices, including dietary habits (ranging from Mediterranean diet to calcium‐rich diets to no particular dietary approach) and physical activity routines (including walking, swimming, pilates, yoga, gymnastics, and no regular exercise).

Eligible participants were people with a confirmed diagnosis of primary osteoporosis defined by a *T*‐score < −2.5 SD (indicating a bone mineral density (BMD) 2.5 standard deviations below that of a healthy young adult reference population, consistent with a diagnosis of osteoporosis) (Kanis 2002); all participants were fully oriented to time, place and person. Subjects were excluded if they had secondary osteoporosis, a *T*‐score above −2.5 SD (indicating osteopenia if BMD is between −1 and −2.5, and normality at BMD ≥ −1) (Kanis 2002), cognitive impairment, linguistic barriers, or declined participation. Interviews were conducted in participants' native languages by trained, native‐speaking researchers with expertise in osteoporosis care and qualitative interviewing.

### 3.2 Data Collection

In Italy, data collection (recruitment and interviews) began in March 2023 and continued until June 2023, whereas in Spain, it took place between March and May 2024. Corpus stabilization was assessed independently for each national group: the Italian corpus reached lexical stability by June 2023, and the Spanish corpus by May 2024, at which point the full cross‐national corpus was considered stable for analysis (Bolasco 2021). Through purposive sampling, all individuals approached in both Italy and Spain agreed to participate, with no dropouts or withdrawals.

Recruitment and interviews were conducted by nursing researchers with academic expertise in the topic. In Italy, interviews were carried out by a female PhD student in Nursing and Public Health (C.T.; RN, MSc), and in Spain by a male Assistant Professor (V.B.‐M.; PhD); both were native speakers and unknown to participants prior to recruitment. During the initial contact, researchers introduced themselves, explained the purpose and procedures of the study, obtained informed consent, administered a short questionnaire to capture participants' sociodemographic and clinical characteristics and then scheduled the interview.

Data were collected through semi‐structured interviews whose guide was developed according to the Middle‐Range Theory of Self‐Care of Chronic Illness (Riegel et al. 2012, 2026) and consisted of 11 open‐ended questions exploring the three dimensions of self‐care maintenance, self‐care monitoring, and self‐care management (Table 1). This structure ensured methodological coherence and allowed participants to articulate their experiences freely while maintaining analytic alignment with the theoretical framework.

**TABLE 1:** Interview guide.

Interview guide		
Dimension explored	Main question	Supporting questions
Self‐care maintenance	What do you do to keep your osteoporosis stable and to feel well?	Who advised you on the actions to take to keep your osteoporosis stable?
	What do you do to manage your osteoporosis therapy?	What do you do to manage any other remedies for osteoporosis?
	What type of diet or nutrition plan are you following for your osteoporosis?	
	What do you do to stay physically active?	
Self‐care monitoring	How do you monitor your osteoporosis?	What measures do you take to keep your osteoporosis under control?
		What makes you realize that something is wrong?
		What do you do to understand if there are problems caused by osteoporosis?
	What do you do to check for side effects of the medications you take for osteoporosis (e.g., gastrointestinal issues)?	
Self‐care management	What do you do when you notice something is wrong related to your osteoporosis?	What do you do when you feel unwell due to osteoporosis?
		What do you do in the presence of symptoms (e.g., postural deformities or height loss) or in the case of complications (e.g., acute pain and fragility fractures of the vertebrae, femur, wrist, humerus, etc.)?
	What do you do if side effects appear from the medications you take for osteoporosis?	
	What makes you think you are managing the disease well?	
	If you encounter problems, whom do you turn to, and what type of advice do you seek?	How often do you consult a clinician?
Free open question	Would you like to add anything else before the end of the interview?	

Interviews were conducted remotely via video calls, using the WhatsApp VoIP system. Participants joined from their homes and were encouraged to choose a quiet and comfortable setting and to express themselves freely. No one other than the participants and researchers was present during data collection. The interview approach encouraged open‐ended responses, with researchers remaining attentive to minimizing leading questions or personal assumptions. Interviews were conducted in the participants' native language to support clarity and openness (Rhisiart et al. 2022) and followed a non‐directive style to encourage reflection (Råheim et al. 2016). Each interview lasted approximately 30 min, and no repeat interviews were conducted.

All interviews were audio‐recorded and transcribed verbatim following established qualitative standards. Audio files and transcripts were anonymised and stored on password‐protected institutional servers accessible only to the research team. Handwritten field notes in both countries were produced during and immediately after interviews. These notes captured general impressions and salient features of individual interviews, providing contextual information to support the analytic process (Mayring 2015).

### 3.3 Data Analysis

To ensure comparability between national contexts while preserving the meaning of participants' narratives, all interviews were translated into English following recommended procedures for cross‐national qualitative research, including forward and backward translation by bilingual experts (Chen and Boore 2010; Cuoco et al. 2022). All translated interviews were merged into a single dataset (corpus) and formatted according to IRaMuTeQ standards (Ratinaud 2024).

At the beginning of each interview section, metadata lines were added. These lines function as labels that tell the software which participant characteristics are associated with each segment (e.g., nationality, sex, age group, and the self‐care domain addressed in that portion of the interview—maintenance, monitoring, or management). This step allows the software to recognize groups and variables without manual coding (Table S1).

To verify the reliability of the corpus for the statistical analysis, we assessed the “lexical balance” of the dataset. Following Bolasco's methodological criteria (2021), a reliable corpus requires: (a) a minimum corpus size of 25,000 occurrences, (b) a Type–Token Ratio (TTR) lower than 20%, indicating adequate lexical redundancy for stable statistical processing, and (c) a proportion of hapax (words cited only once) below 50% to assess the variability of the corpus. These thresholds indicate that the text is sufficiently large, not overly lexically variable, and not dominated by rare words, ensuring that the statistical procedures used in AATD can detect meaningful and stable patterns rather than random fluctuations.

The first analytical step consisted of applying Labbé's intertextual distance (Cortelazzo et al. 2013; Labbé and Labbé 2003). This technique focuses exclusively on word frequency distributions within each national subgroup, enabling the assessment of both intra‐group similarity and inter‐group divergence within the corpus. By comparing these frequency patterns, the index quantifies the relative proximity between the two narrative sets: values closer to 0 indicate highly similar lexical structures, whereas values approaching 1 indicate stronger divergence (Cortelazzo et al. 2013; Labbé and Labbé 2003). This procedure provides an initial descriptive overview of how self‐care discourse differs across national contexts.

The second analytical step was CA, which is a multidimensional statistical technique widely used in lexicometry to study relationships among words and categories. In this study, CA was applied to a contingency structure combining nationality and self‐care dimensions, allowing the analysis of how textual occurrences were differentially associated with these categories through *χ* ^2^ contributions. The factorial map represents this joint structure, in which each modality occupies a position in the space and is surrounded by the vocabulary that contributes most strongly to its definition. This procedure enabled the construction of specific lexical profiles reflecting both cultural context and self‐care domains simultaneously. In addition, CA extracts latent dimensions expressed as factorial axes, which reveal deeper semantic organizations not immediately visible in linear reading. Interpreting these axes supports a hermeneutic understanding of how self‐care meanings are structured across national contexts (Gadamer 2006).

Combining Labbé's intertextual distance with CA provided complementary perspectives: the former quantified the overall lexical divergence between national groups, whereas the latter identified the specific words driving these differences and revealed the semantic structures underlying participants' accounts. Together, these analyses supported a rigorous, data‐driven, and culturally sensitive interpretation of how Italian and Spanish participants articulated their self‐care behaviors in the context of osteoporosis.

### 3.4 Rigor and Reflexivity

The study followed the trustworthiness criteria proposed by Lincoln et al. (1985) to ensure methodological rigor. Credibility was supported through multiple strategies. The interviews were conducted by two native‐speaking researchers and the translation process included forward and back translation by bilingual experts, ensuring semantic and lexical equivalence across language (Chen and Boore 2010; Cuoco et al. 2022). Lexical balance assessment confirmed the adequacy of the corpus for analysis (Bolasco 2021). Credibility was further enhanced by corpus stabilization, ensuring discursive redundancy at the corpus level: in line with lexicometric principles, saturation was therefore considered in terms of corpus redundancy rather than the emergence of manually coded themes (Bolasco 2021). Credibility was also reinforced by participant triangulation, achieved through member checking with a subset of participants, who confirmed that the findings resonated with their lived experiences and accurately reflected their perspectives (Birt et al. 2016). Dependability was ensured by documenting each stage of the analytic process, from corpus construction to statistical modeling, and by adhering to the EMDA framework (Fraire 2009), which provided a consistent methodological pathway. The use of IRaMuTeQ software further supported analytic consistency and reproducibility (Ramos et al. 2019). Reflexive discussions within the team supported this process, allowing interpretations to be continuously questioned and validated (Sanderson et al. 2013). Investigator triangulation further enhanced confirmability, as multiple researchers contributed to coding and interpretation, ensuring that the findings reflected diverse perspectives rather than a single viewpoint (Vivek et al. 2023). In addition, a hermeneutic stance guided the interpretation of latent dimensions: statistical outputs were not treated as mechanical artifacts but were examined considering cultural context and theoretical frameworks (Gadamer 2006). Transferability was promoted by providing detailed descriptions of the sample, the cross‐national settings, and the thematic domains of the analysis. By situating the findings within the Middle‐Range Theory of Self‐Care of Chronic Illness (Riegel et al. 2012, 2026), the study offers insights that may be applied to other chronic conditions and cultural contexts, whereas recognizing the specificities of osteoporosis in Italy and Spain (Stalmeijer et al. 2024). In this way, the findings contribute to advancing culturally sensitive nursing practice and research.

### 3.5 Artificial Intelligence Statement

AI language tools were used to support language editing, grammar checking, and phrasing refinement of certain sections of this manuscript, as well as to enhance the translation of participants' quotations. As none of the authors are native English speakers, these tools helped ensure accurate and contextually appropriate translations, particularly given the presence of colloquial and dialectal expressions in the original texts. All intellectual content, scientific reasoning, interpretation of findings, and conclusions are entirely the product of the authors' work. No AI tool was used to generate, analyze, or interpret data, nor to produce original scientific content. All authors take full responsibility for the accuracy and integrity of the submitted work.

## 4 Results

### 4.1 Sample Characteristics

The study cohort included 40 participants, comprising 38 women and 2 men, with a mean age of 68.6 years (SD = 5.74), equally distributed between Italian (*n* = 20, ID I001–I020) and Spanish (*n* = 20, ID I021–I040) nationalities. A majority of participants (57.5%, *n* = 23) were married or living with a partner, most being retired (67.5%, *n* = 27). Sociodemographic differences were observed between the two national groups. Italian participants showed a higher educational level, with most having completed secondary school (*n* = 9) or holding a university degree (*n* = 1), whereas Spanish participants were predominantly educated to lower secondary school level (*n* = 16). Italian participants more frequently reported having an informal caregiver (63.2%, *n* = 12) compared to Spanish participants (25%, *n* = 5), and predominantly lived with a partner or spouse (68.4%, *n* = 13), whereas Spanish participants showed greater variability in living arrangements, with a notable proportion living alone (35%, *n* = 7) and a higher rate of widowhood (25%, *n* = 5 versus 10.5%, *n* = 2). Regarding income, Spanish participants more frequently reported a good or excellent income level (75%, *n* = 15), whereas Italian participants more commonly reported a sufficient income (52.6%, *n* = 10).

Concerning clinical characteristics, all participants were affected by primary osteoporosis: 34 with postmenopausal osteoporosis and 6 with senile osteoporosis, with diagnoses spanning from 2000 to 2023. Most of the sample (60%, *n* = 24) had a body mass index (BMI) within the normal range (18.5–24.9). Regarding fracture history, 42.5% (*n* = 17) had no fragility fractures, whereas 57.5% (*n* = 23) experienced fractures, predominantly non‐vertebral (65.2%, *n* = 15). Nearly all participants (97.5%, *n* = 39) reported receiving specific osteoporosis treatment, with treatment initiation ranging from 2007 to 2024. Annual follow‐up with Dual Energy X‐ray Absorptiometry (DXA) was performed by all participants, and 92.5% (*n* = 37) also completed the recommended blood tests. Within the sample, 80% (*n* = 32) reported comorbid conditions, and 77.5% (*n* = 31) were taking medications other than those prescribed for osteoporosis.

Regarding lifestyle factors, the majority reported following either a Mediterranean diet (35%, *n* = 14) or a general healthy diet (35%, *n* = 14), typically including a variety of foods such as milk, meat, nuts, eggs, fish, and vegetables, with low sugar content; only 7.5% (*n* = 3) reported focusing specifically on calcium‐rich foods. Unhealthy behaviors, such as smoking and occasional alcohol consumption, were reported by 20% (*n* = 8) of participants each. Meanwhile, 75% (*n* = 30) engaged in regular physical activity. The main sociodemographic and clinical characteristics of the participants are summarized in Table 2.

**TABLE 2:** Participant's characteristics.

ID	Gender	Age	Marital status	Education	Occupation	Fracture, site	Treatment	Physical exercise habits	Principal dietary habits
I01	Female	66–70	Single	High school	Retired		Alendronate; vitamin D		Scarce and inconsistent
I02	Male	61–65	Married	Lower secondary school	Retired		Denosumab; vitamin D		Mediterranean diet
I03	Female	66–70	Married	High school	Housewife		Calcium carbonate + cholecalciferol	Fast walking	No particular diet
I04	Female	55–60	Widowed	Lower secondary school	Unemployed		Vitamin D; calcium carbonate; bisphosphonates; rosuvastatin	Postural exercises	Mediterranean diet
I05	Female	55–60	Widowed	High school	Retired		No treatment	Swimming	Mediterranean diet
I06	Female	66–70	Married	High school	Retired	Yes, left malleolus	Alendronate + cholecalciferol; vitamin D	Yoga; Nordic walking	Mediterranean diet
I07	Female	71–75	Single	Elementary school	Retired	Yes, wrist	Bisphosphonates		No particular diet
I08	Female	66–70	Married	Lower secondary school	Housewife	Yes, vertebral	Denosumab	Swimming	Mediterranean diet
I09	Female	71–75	Married	Lower secondary school	Retired	Yes, vertebral	Alendronate + cholecalciferol; vitamin D	Gymnastics	Eat healthy
I10	Female	55–60	Divorced	Lower secondary school	Manual worker		Bisphosphonates		Healthy mediterranean diet
I11	Female	71–75	Single	Lower secondary school	Retired		Denosumab; vitamin D		No particular diet
I12	Female	76–80	Married	High school	Retired	Yes, left malleolus	Denosumab; vitamin D		Mediterranean diet
I13	Female	66–70	Married	High school	Retired	Yes, vertebral	Denosumab; vitamin D		No particular diet
I14	Female	61–65	Married	High school	Employee		Denosumab; vitamin D	Yoga and walking	No particular diet
I15	Female	61–65	Married	High school	Unemployed		Bisphosphonates; vitamin D	Pilates	Mediterranean diet
I16	Female	61–65	Single	University degree	Employee		Denosumab	Fast walking	Mediterranean diet
I17	Female	55–60	Married	Lower secondary school	Unemployed	Yes, pelvis	Denosumab; vitamin D	Walking and swimming	No particular diet
I18	Female	75–80	Married	Elementary school	Retired	Yes, left and right femurs	Bisphosphonates; vitamin D	Light exercise	No particular diet
I19	Female	71–75	Married	Lower secondary school	Retired		Calcium carbonate + cholecalciferol; vitamin D; denosumab		No particular diet
I20	Female	66–70	Married	High school	Retired		Denosumab	Yoga	Mediterranean diet
S01	Female	55–60	Divorced	Lower secondary school	Employee		Calcium carbonate + cholecalciferol; denosumab	Dancing and walking	Calcium‐rich diet (gruyere cheese, clams, cabbage)
S02	Female	81–85	Widowed	University degree	Retired	Yes, left little finger	Ostein‐hydroxyapatite complex; denosumab	Walk and exercises	Meat, chicken, fish
S03	Female	66–70	Married	Lower secondary school	Retired	Yes, left fibula	Calcium carbonate + cholecalciferol; denosumab	Traveling and walking	No particular diet
S04	Female	61–65	Divorced	Lower secondary school	Employee	Yes, right radius	Calcium carbonate + cholecalciferol; denosumab	Walking and swimming	Mediterranean diet, vegetables, fish, meat, dessert on weekends
S05	Female	66–70	Married	High school	Retired		Ostein‐hydroxyapatite complex; denosumab	Walking	Healthy foods (yogurt, milk, fruit, meat, vegetables, eggs)
S06	Female	75–80	Widowed	Lower secondary school	Retired	Yes, Colles, supracondylar elbow	Vitamin D; denosumab	Walking	Lactose‐free diet
S07	Female	61–65	Married	Lower secondary school	Officer	Yes, ribs	Calcium carbonate + cholecalciferol; vitamin D; betamethasone	Walking 1–1.5 h	Milk and cheese
S08	Female	55–60	Married	University degree	Employee	Yes, vertebral	Denosumab; calcium carbonate + cholecalciferol; alendronate	Pilates and walking	Mediterranean diet, milk, nuts, eggs, fish, and vegetables
S09	Female	71–75	Married	Lower secondary school	Retired		Denosumab; calcifediol monohydrate	Pilates and walking	Healthy diet, milk, nuts, eggs, meat, legumes, and vegetables
S10	Female	66–70	Divorced	Lower secondary school	Retired		Denosumab; calcifediol monohydrate	Pilates and walking	Yogurt, nuts, eggs, fish, vegetables, cheese
S11	Female	66–70	Married	Lower secondary school	Retired	Yes, vertebral	Calcifediol monohydrate; calcium carbonate + cholecalciferol	Walking and country dance	Yogurt, nuts, eggs, fish, vegetables, cheese, pasta
S12	Female	75–80	Widowed	Lower secondary school	Retired	Yes, vertebral	Denosumab; calcifediol monohydrate	Walking	Yogurt, nuts, eggs, fish, vegetables, cheese, fruit, and milk
S13	Female	81–85	Widowed	Lower secondary school	Retired	Yes, shoulder, wrist	Calcifediol monohydrate; olmesartan medoxomil/amlodipine	Tends a small garden	Calcium‐rich milk, blueberries
S14	Female	66–70	Married	High school	Retired	Yes, right tibia and fibula	Calcifediol monohydrate; teriparatide	Walking	Milk, nuts, legumes, rice
S15	Male	61–65	Married	Lower secondary school	Unemployed	Yes, vetrebral	Ostein‐hydroxyapatite complex; vitamin D	Walking	Milk, nuts, legumes, vegetables
S16	Female	61–65	Single	Lower secondary school	Retired	Yes, femur and knee	Ostein‐hydroxyapatite complex; denosumab		Milk, nuts, orange
S17	Female	66–70	Widowed	Lower secondary school	Retired	Yes, vertebral, tibia and fibula	Denosumab; calcium carbonate + cholecalciferol	Walking, gymnastics and stretching	Milk, nuts, orange, vegetables, legumes
S18	Female	71–75	Married	Lower secondary school	Retired	Yes, right olecranon	Denosumab; sodium risedronate	Walking	Milk, nuts, orange, vegetables, fish
S19	Female	61–65	Single	Lower secondary school	Retired		Denosumab; calcifediol monohydrate		Milk, cheese, oranges, nuts, and rice
S20	Female	66–70	Married	Lower secondary school	Retired	Yes, vertebral	Denosumab; calcium carbonate + cholecalciferol	Hiking	Milk, cheese, oranges, nuts, yogurt, legumes

*Note:* Italian participants have IDs from I01 to I20; Spanish participants have IDs from S01 to S20.

Both groups were recruited from public hospital outpatient clinics located in urban settings: the Tor Vergata University Hospital, a large academic hospital situated in the southeastern periphery of Rome, Italy, and the Hospital General Universitari de Castelló in Castellón de la Plana, Spain. The main sociodemographic and clinical characteristics of the participants are summarized in Table 2.

### 4.2 Corpus Description

The corpus shows solid lexical robustness according to Bolasco's methodological criteria (Bolasco 2021). The 27,246 occurrences ensure an adequate textual mass for statistical elaboration, allowing the emergence of stable co‐occurrence patterns and reliable factorial structures. The TTR, calculated as the ratio between the 2065 distinct forms and the total occurrences, was 0.076 (7.6%), a value consistent with large qualitative corpora in which lexical repetition is expected and necessary for robust analysis. Moreover, the corpus contained 812 hapax legomena, corresponding to 2.98% of all occurrences and 39.32% of the total forms. This balance, characterized by a limited percentage of hapax and a sufficiently broad lexical repertoire, indicated that the corpus was neither excessively redundant nor excessively fragmented. Taken together, the TTR, the volume of occurrences, and the distribution of hapax demonstrated that the corpus met Bolasco's criteria for lexical stability and statistical reliability, making it suitable for multidimensional analyses (Table 3).

**TABLE 3:** Lexical balance.

Lexical balance	
Number of texts	120
Number of occurrences	27,246
Number of forms	2065
Number of hapaxes	812 (2.98% of occurrences – 39.32% of forms)

*Note:* The corpus exhibits a solid lexical balance, characterized by a high number of occurrences, a limited number of distinct forms, and a low proportion of hapax legomena. This configuration reflects adequate lexical redundancy and internal coherence of the dataset, supporting the reliability of the subsequent lexicometric analyses.

### 4.3 Labbé's Distance Analysis

The analysis of Labbé's intertextual distance revealed clear patterns of intra‐national lexical cohesion within each subgroup, contrasted with substantial inter‐national divergence between Italian and Spanish narratives. Although Labbé's distance does not rely on universally established cut‐off values, scores closer to 0 indicate highly similar lexical structures, whereas values approaching 1 reflect stronger divergence (Cortelazzo et al. 2013; Labbé and Labbé 2003). The average distance within the Italian subset was 0.262 and within the Spanish subset 0.275. These values showed that participants within each country used similar vocabulary and narrative patterns when describing their experiences of living with osteoporosis, indicating intra‐national coherence. In contrast, the inter‐national average distance between interviews was substantially higher (0.449), indicating greater cross‐national divergence in how participants described self‐care, bodily sensations, and everyday management of the condition (Table 4).

**TABLE 4:** Comparison of intertextual distances.

Comparison	X1	X2	Average distance	Interpretation
Within Italian subset	ITA	ITA	0.262	Moderate similarity within the text
Within Spanish subset	ESP	ESP	0.275	Moderate similarity within the text
Between Italian and Spanish sets	ITA	ESP	0.449	Substantial divergence between texts

*Note:* Average intertextual distance between Italian and Spanish interview subsets (Labbé's distance). Italian and Spanish participants, respectively, used relatively coherent and internally consistent vocabularies when narrating their self‐care experiences. In contrast, the higher distance observed between the Italian and Spanish subsets indicates substantial cross‐national lexical divergence, suggesting that participants from the two countries relied on clearly different linguistic repertoires to describe self‐care in the context of osteoporosis.

Beyond quantifying international lexical distance, the analysis enabled the identification of distinct lexical patterns for each national subgroup. The Italian corpus was characterized by frequent references to diagnosis, clinical monitoring, and pharmacological treatment (Tables S2 and S3). High‐frequency terms included *osteoporosis* (285), *medication* (118), *general practitioner* (43), *therapy* (37), and specific drugs such as *alendronate* (17) and *denosumab* (19). Diagnostic and evaluative terminology (*condition*, 91; *MOC scan*, 35; *check‐up*, 37) appeared consistently. Emotional or experiential terms were comparatively less frequent (*feel*, 88; *afraid*, 5; *hurt*, 3), and references to physical activity were present but less prominent than in the Spanish corpus (*walk*, 56; *exercise*, 21). Dietary vocabulary appeared more limited (*eat*, 26; *milk*, 9; *calcium*, 25).

The Spanish corpus was characterized by higher frequencies of vocabulary related to movement, daily activities, and bodily sensations. Words related to routine physical activity appeared more frequently (*walk*, 125; *exercise*, 53; *run*, 12; *carry*, 15). Dietary references were more numerous and varied (*eat*, 85; *milk*, 35; *cheese*, 21; *calcium*, 39), and emotional vocabulary appeared with higher frequency (*feel*, 150; *hurt*, 62; *afraid*, 24). A distinctive feature of the Spanish corpus was the high frequency of temporal markers (*day*, 110; *time*, 92; *week*, 29). General experiential placeholders such as *thing* were used more often (154 vs. 29 ITA). Reference to falls was far more frequent (*fall*, 81 vs. 22 ITA), and vocabulary describing bodily sensations appeared more explicitly represented (*back*, 56; *hip*, 13; *hurt*, 62). References to healthcare professionals and services were comparatively rare (*general practitioner*, 0; *therapy*, 2; *physiotherapist*, 2).

These patterns describe two distinct lexical configurations that differentiate the Italian and Spanish narratives in terms of vocabulary distribution and thematic emphasis.

### 4.4 Correspondence Analysis (CA)

**FIGURE 1:**
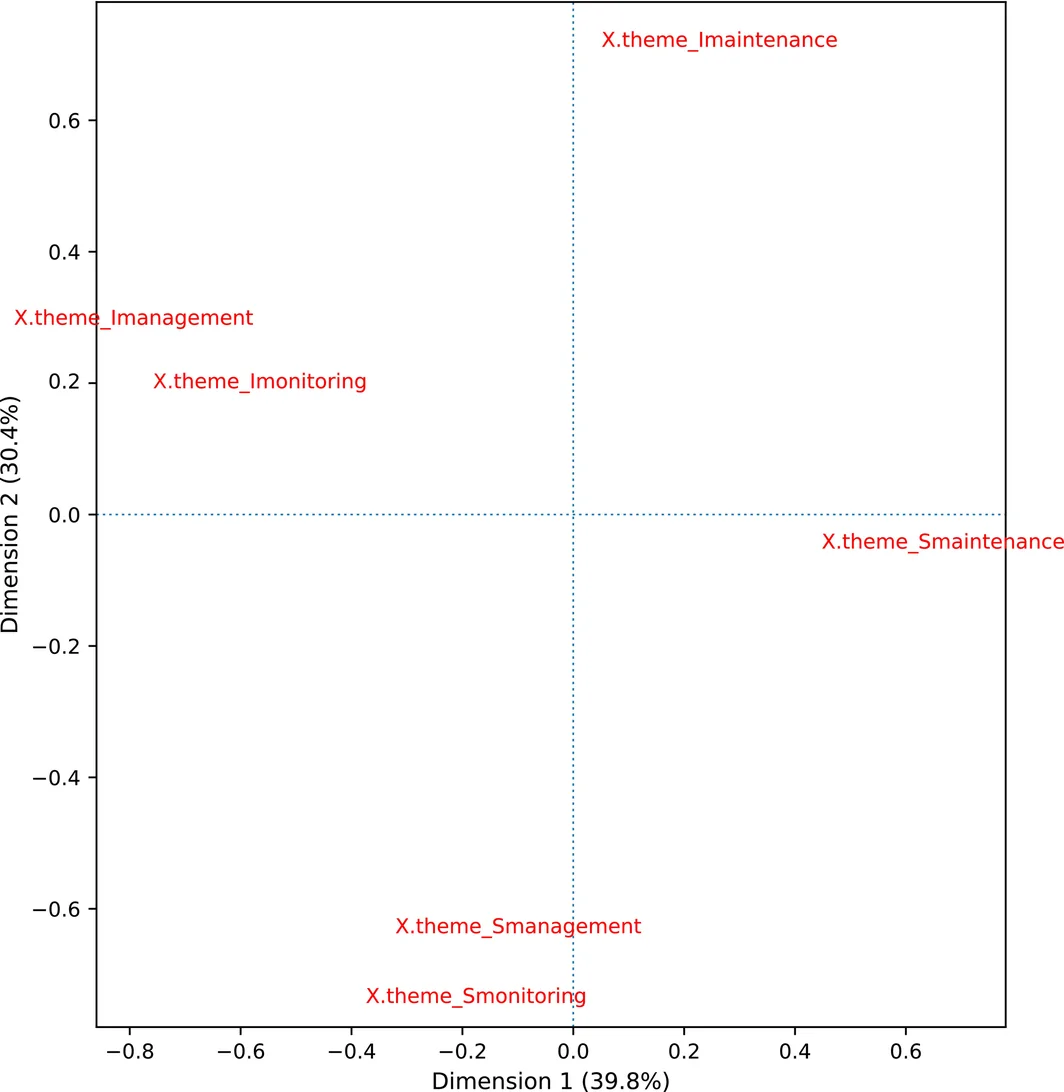
Positioning of self‐care domains by nationality on the factorial plane. Displays the factorial map generated by correspondence analysis, illustrating the positioning of the three self‐care domains, maintenance, monitoring, and management, according to nationality. Each point represents a categorical modality resulting from the combination of self‐care domain and country (Italian or Spanish). The spatial distribution highlights a clear separation between Italian and Spanish narratives along the vertical axis, with Italian modalities clustering in the upper area of the plane and Spanish modalities in the lower area. In parallel, the horizontal axis differentiates self‐care maintenance from the more reactive domains of monitoring and management. This configuration indicates that both cultural context and self‐care domain contribute to structuring participants' discourses, revealing distinct semantic orientations in how self‐care is conceptualized and articulated across countries. I_maintenance, self‐care maintenance in Italian participants; I_management, self‐care management in Italian participants; I_monitoring, self‐care monitoring in Italian participants; S_maintenance, self‐care maintenance in Spanish participants; S_management, self‐care management in Spanish participants; S_monitoring, self‐care monitoring in Spanish participants.

**FIGURE 2:**
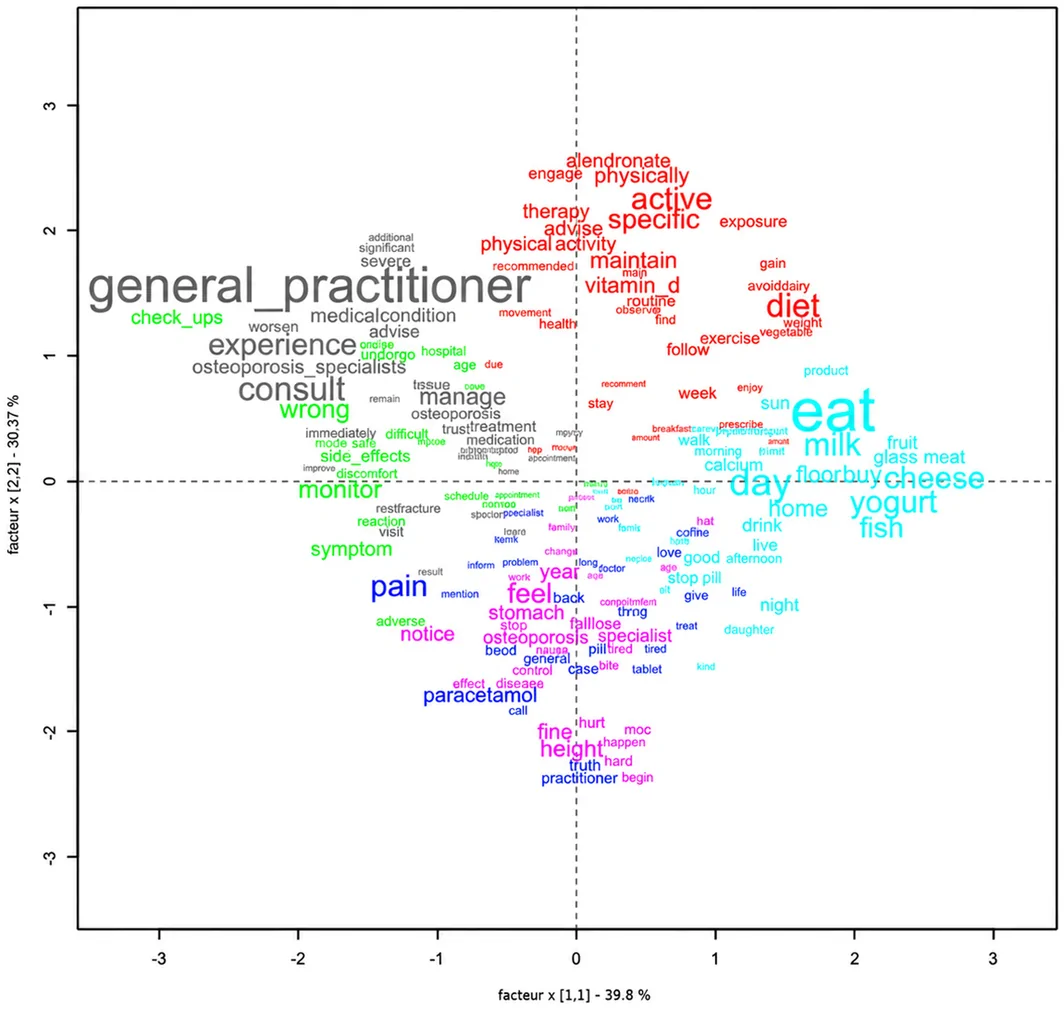
Lexical structuring of self‐care domains by nationality. Presents the correspondence analysis map showing the distribution of lexical items associated with the three self‐care domains (maintenance, monitoring, and management) in Italian and Spanish participants. Words positioned closer to a given self‐care domain are those that contribute most strongly to its lexical characterization based on chi‐square association values. The figure illustrates how Italian and Spanish participants mobilize distinct vocabularies to describe similar theoretical components of self‐care. Italian self‐care domains are primarily associated with a medicalised and institutionally oriented lexicon, whereas Spanish domains are characterized by a more experiential, embodied, and routine‐based vocabulary. The spatial organization of words on the factorial plane reflects underlying semantic structures that differentiate preventive versus reactive self‐care practices and highlight culturally specific ways of interpreting and managing osteoporosis in daily life.

CA was conducted to examine how vocabulary associated with the three theoretical domains of the Middle‐Range Theory of Self‐Care was distributed across the Italian and Spanish interviews. Figure 1 shows a clear structuring of the semantic space according to nationality and the self‐care domain. Italian modalities occupied the upper region of the factorial plane, whereas Spanish modalities clustered in the lower region. The vertical axis differentiated the theoretical domains: maintenance appeared in the right quadrants, whereas monitoring and management were positioned on the left. This distribution indicates lexical differentiation between domains within each national subgroup. Figure 2 illustrates vocabulary associated with each modality. Table 5 synthesizes cross‐national lexical patterns across self‐care domains, whereas the full *χ* ^2^ specificity table is reported in Table S4.

**TABLE 5:** Cross‐national lexical patterns across self‐care domains.

Self‐care domain	Italy—key terms	Spain—key terms
Maintenance	Therapy, prescribe, alendronate, denosumab, treatment, medical, doctor, advice	Eat, milk, cheese, yogurt, calcium, drink, day, morning, week
Monitoring	Exam, test, blood, control, result, osteoporosis	Feel, pain, hurt, notice, symptom, afraid, bother, terrible, emotion
Management	General_practitioner, hospital, specialist, consult, physiotherapist	Rest, stop, routine, daily, stable, life

*Note:* The table highlights clear cross‐national differences in how the three self‐care domains are linguistically articulated. Italian narratives are predominantly characterized by medically and institutionally oriented terms, whereas Spanish narratives emphasize experiential, routine‐based, and embodied vocabulary. Across all domains, these patterns indicate culturally distinct ways of conceptualizing and enacting self‐care in the context of osteoporosis.

#### 4.4.1 Self‐Care Maintenance

The Italian maintenance cluster (red cluster) was characterized by a concentration of vocabulary related to pharmacological treatment and medical supervision. The most associated terms included *diet*, *exposure*, *physical activity*, *therapy*, *prescribe*, *alendronate*, *denosumab*, *treatment*, *medical*, *doctor*, and *advice*. The lexical profile combined medication‐related terminology with references to clinical follow‐up and professionally guided health activities, creating a vocabulary field structured around treatment and organized care practices.

In contrast, the Spanish maintenance cluster (light blue cluster) was dominated by vocabulary related to food intake, daily habits, and temporal routines. Associated terms included *eat*, *milk*, *cheese*, *yogurt*, *calcium*, and *drink*, together with temporal markers such as *day*, *morning*, and *week*. The lexical field consisted primarily of everyday actions and time‐referenced vocabulary, forming a cluster organized around routine domestic practices and recurring daily activities.

#### 4.4.2 Self‐Care Monitoring

The Italian monitoring cluster (green cluster) was dominated by technical and diagnostic vocabulary, including *exam*, *test*, *blood*, *control*, *result*, and *osteoporosis*. The lexical profile concentrated on terminology associated with medical testing, measurement, and formal clinical evaluation, forming a vocabulary field structured around diagnostic procedures and biomedical assessment.

In contrast, the Spanish monitoring cluster (violet cluster) was characterized by vocabulary describing bodily sensations and subjective states. Central terms included *feel*, *pain*, *hurt*, *notice*, *symptom*, *afraid*, and *bother*. The lexical field consisted of words referring to sensory perception, discomfort, and emotional responses, creating a cluster organized around experiential descriptions of bodily change.

#### 4.4.3 Self‐Care Management

The Italian management cluster (gray cluster) was characterized by vocabulary related to healthcare services and professional consultation. Associated terms included *general practitioner*, *hospital*, *specialist*, *consult*, *physiotherapist*, and *manage*. The lexical profile concentrated on references to clinical appointments, healthcare institutions, and specialist involvement, forming a vocabulary field structured around formal healthcare interactions.

In contrast, the Spanish management cluster (blue cluster) was dominated by vocabulary related to daily regulation and activity adjustment. Terms included *rest*, *stop*, *routine*, *daily*, *stable*, and *life*. The lexical field consisted primarily of words describing pacing, rest, and modifications of everyday activities, forming a cluster organized around routine management of daily functioning.

### 4.5 Latent Dimensions

Two latent dimensions emerged from the CA, each capturing structured oppositions in the lexical organization of maintenance, monitoring, and management. These dimensions reveal how the vocabulary of self‐care is organized around contrasting semantic poles.

Factor 1, Called “Everyday self‐care behaviors” (Explained Variance: 39.80%), described a continuum between two lexical configurations of self‐care. The left pole clustered vocabulary associated with healthcare interaction and biomedical management, including *general practitioner*, *consult*, *check‐up*, *monitor*, *medication*, *symptom*, *side effects*, and *pain*, expressing a model of self‐care grounded in biomedical control, professional follow‐up, and pharmacological oversight. The right pole grouped terms related to diet and daily practices, such as *eat*, *diet*, *yogurt*, *cheese*, *milk*, *vitamin D*, *exercise*, and *day*, indicating a self‐care model embedded in everyday routines, nutritional choices, and autonomous behavioral regulation. This axis, therefore, captures the contrast between a functional–institutional conception of self‐care, mediated by the healthcare system, and a lifestyle‐oriented conception rooted in the rhythms of daily life.

Factor 2, called “Barriers and facilitators to self‐care” (Explained variance: 30.37%), captured a functional–vulnerability continuum, contrasting lexical fields associated with resources that support self‐care and vocabulary describing symptom‐related and emotional burdens.

The upper pole grouped terms such as *active*, *exercise*, *sunlight*, *vitamin D*, *general practitioner*, and *check‐up*, reflecting physical capacity, proactive engagement, and structured medical follow‐up. The lower pole included words such as *pain*, *hurt*, *fall*, *weak*, *feel*, *paracetamol*, and *bad*, representing vocabulary associated with discomfort, perceived weakness, and vulnerability.

These dimensions highlight different approaches to articulating self‐care, organized around contrasts between clinical and everyday practices and between functional capacity and vulnerability.

## 5 Discussion

This cross‐national study explored and compared self‐care narratives among Italian and Spanish PwOP using an EMDA‐based analytical approach to qualitative textual data. In line with the study aim, the analysis examined how self‐care was discursively constructed across two comparable national contexts, allowing culturally embedded differences in attitudes and practices to emerge and enabling cross‐cultural comparability.

Overall, although Labbé's intertextual distance does not rely on fixed cut‐off thresholds, the relative contrast between intra‐ and inter‐national values indicates a clear pattern of cross‐national divergence. Consistent with its established use in multidimensional textual research, Labbé's distance is interpreted comparatively rather than as a rigid classificatory measure (Cortelazzo et al. 2013; Labbé and Labbé 2003). Each group exhibited moderate intra‐national lexical similarity, whereas substantially greater divergence emerged between Italian and Spanish narratives. These findings suggest that participants draw on distinct cultural and epistemic frameworks when interpreting and enacting self‐care in the context of osteoporosis (Kleinman 1988; Osokpo and Riegel 2021; Riegel et al. 2012).

Italian participants' narratives portrayed self‐care as an episodic, event‐driven, and medically mediated process centred on healthcare institutions and professional guidance. The discourse was characterized by a predominantly pharmacological and diagnostic orientation, with self‐care described as externally guided rather than autonomously enacted. References to professional interactions and technical language were particularly salient, reinforcing a model of care anchored in healthcare supervision rather than personal adaptation. This orientation may partly reflect structural features of the Italian healthcare system, where primary care is regionally fragmented and general practitioners act as gatekeepers to specialized services (Sánchez‐Sagrado 2018). Previous research on Italian PwOP has similarly documented how trust in healthcare providers enhances motivation, whereas perceived neglect generates frustration (Tormen et al. 2025), a relational pattern also observed in Italian heart failure self‐care (Cocchieri et al. 2015).

In contrast, Spanish participants' narratives more frequently portrayed self‐care as embodied, routine‐based, and emotionally expressive, framed within everyday life rather than primarily through medical mediation. A distinctive feature of the Spanish discourse was its temporal orientation: self‐care was narrated as a continuous practice integrated into daily and weekly routines, rather than as episodic responses to medical events. Spanish accounts of dietary practices were detailed and spontaneous, contrasting with findings from the Spanish validation of the Self‐Care of Chronic Illness Inventory, where “special diet” loaded onto illness‐related behaviors, suggesting that older adults may perceive dietary adaptation as an imposed necessity rather than a voluntary healthy habit (Hernández‐Padilla et al. 2024). This discrepancy may reflect methodological differences: open‐ended narratives in our study may have captured the personal meanings and integration of dietary practices that can remain less visible in quantitative assessment. Spanish narratives also emphasized experiential and pragmatic adaptation, with vocabulary centred on bodily perception and daily adjustment rather than technical surveillance or professional guidance. This pattern may reflect an interplay between sociocultural norms shaping emotional expressiveness (Epstein and Borrell i Carrió 2001) and a healthcare context that has increasingly supported patient autonomy and collaborative self‐management through structured health education in primary care (Busquets et al. 2012; Durán‐Gómez et al. 2023).

These findings are consistent with the literature, indicating that, nonetheless, the theoretical constructs of self‐care maintenance, monitoring, and management may transcend cultural boundaries (De Maria et al. 2021). Self‐care behaviors can be influenced by cross‐cultural dynamics, individual beliefs, and contextual factors (Cittadini et al. 2022; Jaarsma et al. 2013; Osokpo and Riegel 2021; Tedesco, Bernalte‐MartÍ, Tormen, et al. 2025; Tormen et al. 2025; Zeng et al. 2023).

Two fundamental tensions emerged from the latent dimensions, structuring how PwOP experience and articulate self‐care. The first reflects a functional‐institutional versus everyday‐embedded polarity: chronic illness management may be experienced either as dependent on healthcare institutions, anchored in clinical oversight, pharmacological control, and professional guidance, or incorporated into the everyday rhythms through nutrition, movement, and autonomous routines. This distinction highlights divergent epistemic orientations regarding whether the healthcare system or everyday existence constitutes the primary locus of control. The second tension articulates a symptomatic‐emotional polarity, capturing the ongoing negotiation between resources that enable self‐care and vulnerabilities that constrain it. This aligns with evidence showing that self‐care adequacy depends on the balance between barriers and facilitators (Bayliss 2003; Bernalte‐Martí et al. 2026; Paolucci et al. 2016; Riegel et al. 2019). These dimensions suggest that cultural context plays a structuring role in how PwOP position themselves within these polarities.

Although this study was not designed to correlate linguistic patterns with sociodemographic or clinical variables, several descriptive alignments warrant attention. Spanish participants frequently named foods and diets related to osteoporosis management, whereas Italian participants more commonly referenced generic dietary approaches such as the Mediterranean diet. Similarly, Spanish participants reported more frequent engagement in specific physical activities. These patterns corroborated the CA findings, emphasizing specific behavioral aspects of self‐care over medicalized approaches. Paradoxically, the Spanish sample reported a higher prevalence of fragility fractures compared to Italian participants. A possible explanation relates to diagnostic timing. Given Spain's lower DXA availability (15.5 vs. 23.5 units per million population) and substantially longer waiting times (180 vs. 90 days) (Willers et al. 2022), fractures may have occurred before osteoporosis diagnosis, with the fracture itself serving as the sentinel event triggering assessment.

These findings reveal that Italian and Spanish participants do not simply perform different behaviors, but they rely on culturally patterned ways of interpreting and organizing their experiences; these differences may reflect that, despite shared universal healthcare principles, the Mediterranean macro‐region has developed distinct organizational models shaping healthcare delivery and patient experiences (Giarelli 2021). Comparative research on mental health management in Italian and Spanish primary care has confirmed this pattern, identifying how shared healthcare principles coexist with nationally distinct organizational configurations and professional cultures, influencing how patients and providers navigate chronic illness management (Giosa 2024).

These nationally distinct organizational configurations may also shape the extent to which PwOP are exposed to structured health promotion programmes and supported in developing autonomous self‐management skills. The predominantly pharmacological and appointment‐centred orientation observed in Italian PwOP narratives is consistent with patterns documented across Italian chronic illness populations, where medication adherence and attendance at scheduled visits were the most performed maintenance behaviors, physical activity and stress management the least performed, monitoring was largely structured around diagnostic examinations rather than autonomous symptom surveillance, and management behaviors were predominantly triggered by professional consultation (Ausili et al. 2017; Di Nitto et al. 2022; Napolitano et al. 2025; Vellone et al. 2020). This pattern may partly reflect the fragmented landscape of health promotion in Italy, where innovative initiatives (including regional walking programmes, thermal spa‐based interventions, and participation in European collaborative research networks) coexist with strong regional autonomy and persistent territorial inequalities that limit equitable access to structured programmes (Illario et al. 2015; Mantineo and Ignatenko 2026; Noviello et al. 2026; Pagani et al. 2023). Poor care continuity, identified as a primary barrier to self‐care engagement in osteoporosis, may further reinforce reliance on episodic clinical encounters rather than sustained autonomous self‐management (Bernalte‐Martí et al. 2026). In contrast, the more embodied and routine‐based self‐care orientation emerging from Spanish narratives aligns with the notably high monitoring scores and adequate management scores documented in Spanish patients with heart failure and multiple chronic conditions, where contacting healthcare providers remained the most performed management behavior but self‐care was overall more integrated (Antonio‐Oriola et al. 2022; Durán‐Gómez et al. 2023; Juárez‐Vela et al. 2021), a pattern consistent with a primary care model that has increasingly promoted patient autonomy and collaborative self‐management through structured health education (Busquets et al. 2012; Durán‐Gómez et al. 2023). Taken together, these convergences may suggest that the discursive patterns identified in this study reflect broader culturally and structurally embedded orientations toward self‐care that appear to extend beyond osteoporosis, pointing to the potential value of culturally responsive and system‐aware approaches to self‐care education and support in this population.

### 5.1 Strengths and Limitations

Institutional and experiential variations may shape how PwOP conceptualize and enact self‐care, with important practical implications. For Italian PwOP, interventions may benefit from encouraging the internalization of self‐care into daily routines, fostering autonomy and proactive management while leveraging healthcare professionals as supportive resources. For Spanish PwOP, acknowledging bodily sensations, emotional awareness, and routine‐based strategies may help clinicians integrate subjective experiences into responsive care pathways focused on fracture prevention. Tailoring self‐care education to these culturally anchored orientations may enhance engagement, adherence, and communication. Framed within the Middle‐Range Theory of Self‐Care of Chronic Illness (Riegel et al. 2012, 2026), such tailoring should address self‐care behaviors in ways that resonate with local meanings of guidance and autonomy.

The present study does not aim to support statistical generalization. Within a multidimensional textual framework, the analytical value of the study lies in the structural adequacy of the corpus and in its capacity to generate stable and interpretable discursive patterns, rather than in participant number per se. This study acknowledges that participants' narratives are shaped by multiple contextual and individual factors. Within a multidimensional textual perspective, such variability is not controlled for analytically but is considered an integral component of the discursive configurations identified. The findings should therefore be interpreted as situated patterns of meaning‐making, rather than as expressions attributable to specific individual variables.

Moreover, this study introduces methodological and substantive strengths. First, it applies an innovative EMDA‐based approach not previously used in osteoporosis self‐care research, converting textual variation into measurable patterns while enhancing rigor, transparency, and cross‐cultural comparability. This method reconciles qualitative depth with quantitative precision, providing a reproducible framework that reveals culturally embedded aspects that may remain less visible in traditional qualitative approaches. Second, the analysis operates at multiple levels, capturing intra‐national lexical cohesion and inter‐national divergence through macro‐level intertextual distance and micro‐level semantic structuring, thereby strengthening cross‐national comparison. Finally, findings align with the Middle‐Range Theory of Self‐Care of Chronic Illness (Riegel et al. 2012, 2026), demonstrating theoretical coherence while revealing how cultural context shapes not only vocabulary but also how PwOP position themselves within fundamental polarities of chronic illness management.

However, this study also presents some limitations.

First, from an epistemological perspective, this multidimensional statistical approach does not fit neatly within conventional qualitative, quantitative, or mixed‐methods designs. The analytical focus is placed on corpus‐level textual structures rather than on manually coded themes or illustrative quotations. For this reason, some reporting criteria commonly adopted in qualitative research and in qualitative checklists (e.g., number of coders, codebooks, or quotation‐based theme development) are not fully applicable. This points to a broader methodological gap and suggests the need for study designs and reporting frameworks specifically tailored to corpus‐based, multidimensional textual analysis. Future methodological work may help clarify the epistemological positioning and quality criteria of this approach.

Another methodological consideration concerns the interpretation of Labbé's intertextual distance. This index does not rely on universally standardized cut‐off values and is not intended as an inferential or classificatory measure. Rather, it provides a descriptive indication of relative lexical similarity or divergence between textual corpora (Labbé and Labbé 2003). As a result, its interpretive value lies primarily in comparative patterns observed within and between groups, rather than in absolute thresholds (Bolasco 2021; Cortelazzo et al. 2013). To address this limitation, Labbé's distance was not used in isolation but was integrated with CA, allowing lexical divergence to be further explored, structured, and theoretically interpreted. This combined approach strengthens the robustness of the findings by situating descriptive distance measures within a multivariate and interpretive analytical framework. Although the integration of AATD and Labbé's intertextual distance offered a structured and replicable framework for comparing self‐care discourses across national contexts, some limitations must be acknowledged. Fully automated analysis may reduce interpretive nuance, overlooking subtle contextual cues, emotional undertones, or culturally specific expressions that manual coding could capture. The use of self‐reported narratives introduces the possibility of recall bias, social desirability, or selective emphasis. Participants may have under‐ or over‐reported certain behaviors depending on their beliefs, perceived expectations, or emotional states. In addition, the study focused exclusively on narratives elicited in interview contexts; observations of everyday practices or multimodal data could provide complementary insights into how self‐care is enacted beyond verbal accounts. Additionally, the study was conducted in two convenience‐selected hospital outpatient contexts in Italy and Spain, which, despite cultural and linguistic differences, share structural similarities, limiting exploration of more diverse settings. Future research could expand to varied cultural and clinical environments and integrate manual thematic analysis to enrich interpretation.

Finally, the use of video calls as the sole data collection modality may have excluded older adults with limited access to or familiarity with digital technologies. As a result, the sample may have disproportionately reflected the perspectives of those with sufficient technological competence, potentially limiting the diversity of experiences captured and the transferability of findings to populations with lower digital literacy.

The present study would benefit from larger and more diverse corpora in order to examine the stability and transformation of discursive configurations across contexts. In a multidimensional textual perspective, increasing sample, and so, corpus size allows for a more articulated exploration of variability rather than for statistical generalization. In fact, the sample size, whereas appropriate for qualitative lexicometric analysis, may limit the stability of discursive configurations, and the exclusive reliance on qualitative textual data does not allow for the quantification of self‐care behaviors or for direct comparison with standardized self‐care scores across countries and conditions.

### 5.2 Implication for Nursing Practice

The cross‐national differences observed in self‐care discourse highlight the need for culturally responsive approaches to osteoporosis management. For clinical practice, the findings suggest that self‐care education should not be delivered as a universal protocol but adapted to patients' discursive orientations toward health and illness. In contexts where self‐care is framed primarily through institutional and pharmacological guidance, clinicians may need to actively support the translation of medical prescriptions into sustainable daily routines, fostering patient autonomy while maintaining therapeutic alliance. Conversely, in contexts where self‐care is embedded in experiential and routine‐based practices, clinical encounters may benefit from integrating patients' bodily perceptions and emotional narratives into structured prevention strategies, particularly regarding fracture risk and symptom monitoring. Recognizing these orientations can improve communication, adherence, and shared decision‐making.

In the specific contexts examined, this implies that health education for Italian PwOP may need to prioritize the development of autonomous self‐management skills and the integration of medical guidance into daily routines, whereas for Spanish PwOP, interventions may focus on reinforcing structured monitoring and fracture risk awareness within an already embodied self‐care orientation.

For research, the study demonstrates the importance of incorporating culturally sensitive textual and narrative analysis into self‐care investigation. The narrative method adopted in this study is particularly valuable in this regard, as it reveals the cultural frameworks through which people make sense of their condition, offering a layer of interpretive depth that may complement standardized assessments and may prove essential for designing interventions that are not only clinically appropriate but also culturally resonant. Future studies should explore how discursive patterns interact with socioeconomic, clinical, and health system variables, and whether similar tensions between institutional and everyday self‐care emerge in other chronic conditions. Longitudinal and mixed‐method designs could clarify how these discursive frameworks evolve over time and influence behavioral outcomes. Additionally, integrating textual analysis with standardized self‐care measures, such as the Self‐Care of Chronic Illness Inventory (SC‐CII) (Riegel et al. 2018), may provide complementary insights into the relationship between discursive constructions and behavioral dimensions of self‐care, including maintenance, monitoring, and management. Such approaches may contribute to a more comprehensive understanding of self‐care while preserving the distinct epistemological contribution of each method. Moreover, future research may integrate multidimensional textual analysis with condition‐specific self‐care instruments to explore the relationships between discursive constructions and measurable behavioral dimensions of self‐care. Within EMDA, quantitative variables can be incorporated as illustrative or active variables, allowing the examination of their associations with emergent discursive configurations. Such integration aligns with the exploratory nature of multidimensional textual analysis and mixed‐method designs, enabling the investigation of how self‐care is both constructed in discourse and expressed in practice. However, this integration represents an extension of the analytical framework, aimed at enriching interpretation.

For education, the results underscore the need to prepare healthcare professionals to recognize diverse epistemologies of self‐care. Training programmes in nursing and allied health should include competencies in narrative listening, cultural interpretation, and communication strategies that bridge biomedical knowledge with patients' lived experiences. Teaching self‐care as a relational and contextual practice, rather than a purely behavioral prescription, may enhance professionals' ability to tailor interventions.

At the organizational level, the findings suggest that healthcare systems should design self‐care support structures that acknowledge variability in how patients integrate medical guidance into daily life. Policies promoting patient education, community‐based prevention, and interdisciplinary collaboration should be flexible enough to accommodate culturally patterned approaches to autonomy, institutional trust, and experiential regulation. Embedding culturally responsive self‐care frameworks into organizational models may strengthen chronic illness management and patient engagement.

## 6 Conclusion

This study revealed clear cross‐cultural differences in how PwOP narrate self‐care, with Italian participants emphasizing a medicalised, professionally guided approach, and Spanish participants describing a more autonomous, routine‐based, and emotionally expressive model. These divergent discourses reflect cultural orientations as well as relationships with healthcare systems and perceptions of personal agency, providing a meaningful focus for clinical practice and the potential for tailored interventions. Recognizing these culturally patterned ways of conceptualizing self‐care may inform the development of flexible educational and supportive interventions that are adaptable not only across different national contexts, but also across healthcare systems where similar orientations toward medical authority or personal autonomy may be present. The integration of Labbé's intertextual distance and CA enabled a structured comparison of lexical patterns, showing internal cohesion within national groups and divergence between them, whereas CA highlighted distinctions between preventive and reactive dimensions of self‐care. Future research combining computational and manual thematic analyses could enrich interpretive depth, support triangulation, and strengthen qualitative insights in cross‐cultural health research.

## 7 Clinical Resources

- Self‐Care Measures. *Self‐care of chronic illness instruments and resources*. https://self‐care‐measures.com/.
- IRaMuTeQ: *Interface de R pour les Analyses Multidimensionnelles de Textes et de Questionnaires*. https://pratinaud.gitpages.huma‐num.fr/iramuteq‐website/.
- Royal Osteoporosis Society. *Resources for practice nurses*. https://theros.org.uk/healthcare‐professionals/courses‐and‐cpd/osteoporosis‐resources‐for‐primary‐care/practice‐nurses/.
- International Osteoporosis Foundation. *Our network*. *Fracture Liaison Services (FLS)* https://www.osteoporosis.foundation/our‐network.

## Funding

This work was supported by Universitat Jaume I (grant number: UJI‐2024‐20).

## Ethics Statement

Ethical approval was granted by the Independent Ethics Committee of Policlinico di Tor Vergata on December 2, 2022 (registration number 211.22) and by the Independent Ethics Committee of Hospital General Universitario de Castelló on April 24, 2023 (registration number 5/2023). Before any study procedure or any data collection, participants gave written and formal informed consent with an ethically approved informed consent form.

## Conflicts of Interest

The authors declare no conflicts of interest.

## Supporting information


**Table S1:** Structure and meaning of metadata lines used for corpus segmentation and lexicometric analysis.

**Table S2:** Lexical profile for the Italian subsamples.

**Table S3:** Lexical profile for the Spanish subsample.

**Table S4:** Chi‐square lexical specificity by nationality and self‐care domain. This table reports the chi‐square specificity values of lexical forms significantly associated with each nationality × self‐care domain modality in the correspondence analysis. It identifies the vocabulary statistically overrepresented in maintenance, monitoring, and management across Italian and Spanish interviews, highlighting the lexical contributions driving cross‐national differentiation.

## Data Availability

The data that support the findings of this study are available from the corresponding author upon reasonable request.
