# Supplementary material for: Self‐Care Behaviors in Osteoporosis: A Cross‐Cultural Study Using Multidimensional Analysis of Textual Data
Source: J Nurs Scholarsh. 2026 Sep 14;58(5):e70134. doi: 10.1111/jnu.70134 (PMC13575457; doi:10.1111/jnu.70134)
Supplement: Supplementary file 1 [file JNU-58-0-s001.docx]

Table S3: Lexical profile for the Spanish subsample

**Supplementary material**

| **Word** | **F** | **Word** | **F** | **Word** | **F** | **Word** | **F** | **Word** | **F** | **Word** | **F** |
| --- | --- | --- | --- | --- | --- | --- | --- | --- | --- | --- | --- |
| *thing* | 154 | *tire* | 27 | *fish* | 14 | *bother* | 10 | *breakfast* | 7 | *constantly* | 3 |
| *feel* | 150 | *practitioner* | 27 | *stretch* | 14 | *pass* | 10 | *honestly* | 7 | *attend* | 3 |
| *osteoporosis* | 129 | *find* | 26 | *health* | 13 | *place* | 10 | *surgery* | 7 | *exposure* | 3 |
| *year* | 129 | *normal* | 26 | *age* | 13 | *small* | 10 | *condition* | 6 | *check_up* | 3 |
| *Osteoporosis specialist* | 129 | *avoid* | 25 | *understand* | 13 | *calm* | 10 | *medical* | 6 | *past* | 3 |
| *walk* | 125 | *fracture* | 24 | *special* | 13 | *jump* | 10 | *activity* | 6 | *dentist* | 3 |
| *pain* | 120 | *denosumab* | 24 | *recommend* | 13 | *prefer* | 10 | *physical* | 6 | *clinic* | 3 |
| *day* | 110 | *careful* | 24 | *important* | 13 | *terrible* | 10 | *advise* | 6 | *level* | 3 |
| *time* | 92 | *end* | 24 | *hip* | 13 | *glass* | 10 | *routine* | 6 | *density* | 3 |
| *eat* | 85 | *afraid* | 24 | *stand* | 13 | *travel* | 10 | *hope* | 6 | *Osteoporosis specialists* | 2 |
| *fall* | 81 | *wear* | 24 | *effect* | 13 | *turn* | 10 | *immediately* | 6 | *therapy* | 2 |
| *care* | 76 | *leave* | 23 | *foot* | 13 | *sleep* | 10 | *aware* | 6 | *undergo* | 2 |
| *medication* | 75 | *swim* | 23 | *coffee* | 13 | *inject* | 10 | *regular* | 6 | *lifestyle* | 2 |
| *bone* | 69 | *sit* | 23 | *house* | 13 | *shoe* | 10 | *learn* | 6 | *require* | 2 |
| *life* | 65 | *daughter* | 23 | *medicine* | 13 | *monitor* | 9 | *situation* | 6 | *healthy* | 2 |
| *hurt* | 62 | *lose* | 22 | *relax* | 13 | *advice* | 9 | *low* | 6 | *approach* | 2 |
| *live* | 60 | *diagnose* | 21 | *arm* | 13 | *discomfort* | 9 | *sister* | 6 | *moc_scans* | 2 |
| *bite* | 57 | *cheese* | 21 | *call* | 13 | *difficult* | 9 | *sport* | 6 | *system* | 2 |
| *back* | 56 | *floor* | 21 | *god* | 13 | *early* | 9 | *return* | 6 | *self_care* | 2 |
| *test* | 55 | *explain* | 20 | *suppose* | 13 | *trust* | 9 | *adverse* | 6 | *osteopenia* | 2 |
| *problem* | 54 | *begin* | 20 | *height* | 13 | *fear* | 9 | *strengthen* | 6 | *difficulty* | 2 |
| *follow* | 53 | *sun* | 19 | *anymore* | 13 | *read* | 9 | *mother* | 6 | *develop* | 2 |
| *exercise* | 53 | *case* | 19 | *buy* | 13 | *rest* | 9 | *hospital* | 5 | *factor* | 2 |
| *move* | 50 | *woman* | 18 | *vitamin_d* | 12 | *bring* | 9 | *improve* | 5 | *yoga* | 2 |
| *good* | 50 | *affect* | 18 | *aspect* | 12 | *climb* | 9 | *recommendation* | 5 | *severe* | 1 |
| *work* | 48 | *put* | 18 | *negative* | 12 | *refer* | 9 | *suffer* | 5 | *significant* | 1 |
| *disease* | 48 | *limit* | 18 | *complication* | 12 | *point* | 9 | *cautious* | 5 | *management* | 1 |
| *home* | 48 | *knee* | 18 | *mention* | 12 | *love* | 9 | *center* | 5 | *worsen* | 1 |
| *start* | 47 | *lie* | 18 | *forget* | 12 | *simple* | 9 | *menopause* | 5 | *lead* | 1 |
| *long* | 47 | *night* | 18 | *half* | 12 | *depend* | 9 | *balance* | 5 | *physically* | 1 |
| *notice* | 46 | *pool* | 18 | *food* | 12 | *strange* | 9 | *precaution* | 5 | *patient* | 1 |
| *stop* | 46 | *continue* | 17 | *run* | 12 | *solution* | 9 | *show* | 5 | *healthcare* | 1 |
| *break* | 45 | *positive* | 17 | *fruit* | 12 | *grind* | 9 | *minute* | 5 | *plan* | 1 |
| *diet* | 44 | *hard* | 17 | *pilates* | 12 | *body* | 8 | *period* | 5 | *relate* | 1 |
| *pill* | 43 | *bed* | 17 | *osteoarthritis* | 12 | *appointment* | 8 | *dental* | 5 | *beneficial* | 1 |
| *person* | 41 | *kind* | 17 | *symptom* | 11 | *support* | 8 | *strong* | 5 | *challenge* | 1 |
| *bad* | 41 | *moc* | 17 | *prevent* | 11 | *consultation* | 8 | *dairy* | 5 | *femur* | 1 |
| *treatment* | 40 | *side_effects* | 16 | *wait* | 11 | *instruction* | 8 | *specific* | 4 | *face* | 1 |
| *month* | 39 | *active* | 16 | *easy* | 11 | *vegetable* | 8 | *consult* | 4 | *fortunately* | 1 |
| *calcium* | 39 | *leg* | 16 | *check* | 11 | *side* | 8 | *wrong* | 4 | *rely* | 1 |
| *ago* | 37 | *weight* | 16 | *today* | 11 | *wake* | 8 | *high* | 4 | *benefit* | 1 |
| *stay* | 36 | *stomach* | 16 | *meat* | 11 | *product* | 8 | *spine* | 4 | *proper* | 1 |
| *morning* | 36 | *moment* | 16 | *village* | 11 | *big* | 8 | *cholesterol* | 4 | *General practitioner* | 0 |
| *give* | 35 | *stair* | 16 | *son* | 11 | *tooth* | 8 | *professional* | 4 | *seek* | 0 |
| *milk* | 35 | *hand* | 16 | *talk* | 11 | *g* | 8 | *main* | 4 | *alendronate* | 0 |
| *blood* | 33 | *issue* | 15 | *hit* | 11 | *bicycle* | 8 | *implant* | 4 | *engage* | 0 |
| *prescribe* | 32 | *maintain* | 15 | *due* | 10 | *movement* | 7 | *play* | 4 | *additional* | 0 |
| *doctor* | 32 | *stable* | 15 | *visit* | 10 | *diagnosis* | 7 | *reaction* | 4 | *include* | 0 |
| *drink* | 32 | *remember* | 15 | *receive* | 10 | *remain* | 7 | *decide* | 4 | *ensure* | 0 |
| *happen* | 31 | *paracetamol* | 15 | *result* | 10 | *daily* | 7 | *usual* | 4 | *well_being* | 0 |
| *general* | 31 | *spend* | 15 | *friend* | 10 | *schedule* | 7 | *gain* | 4 | *specialize* | 0 |
| *injection* | 30 | *dance* | 15 | *inform* | 10 | *recently* | 7 | *experience* | 3 | *initially* | 0 |
| *manage* | 29 | *carry* | 15 | *part* | 10 | *mind* | 7 | *check_ups* | 3 | *significantly* | 0 |
| *week* | 29 | *afternoon* | 15 | *treat* | 10 | *gymnastics* | 7 | *moc_scan* | 3 | *adjust* | 0 |
| *fine* | 29 | *yogurt* | 15 | *limitation* | 10 | *family* | 7 | *risk* | 3 | *reassure* | 0 |
| *tablet* | 28 | *enjoy* | 14 | *attention* | 10 | *progress* | 7 | *concern* | 3 | *t_score* | 0 |
| *truth* | 28 | *barrier* | 14 | *pay* | 10 | *great* | 7 | *simply* | 3 |  |  |
| *change* | 27 | *child* | 14 | *fact* | 10 | *step* | 7 | *clear* | 3 |  |  |
| *hour* | 27 | *control* | 14 | *husband* | 10 | *sign* | 7 | *specialist* | 3 |  |  |
| *worry* | 27 | *physiotherapist* | 14 | *young* | 10 | *reach* | 7 | *prevention* | 3 |  |  |

Note: F stands for Frequency
