# Supplementary material for: Self‐Care Behaviors in Osteoporosis: A Cross‐Cultural Study Using Multidimensional Analysis of Textual Data
Source: J Nurs Scholarsh. 2026 Sep 14;58(5):e70134. doi: 10.1111/jnu.70134 (PMC13575457; doi:10.1111/jnu.70134)
Supplement: Supplementary file 2 [file JNU-58-0-s002.docx]

**Supplementary material**

***Table S4:*** *Chi****-square lexical specificity by nationality and self-care domain***
This table reports the chi-square specificity values of lexical forms significantly associated with each nationality × self-care domain modality in the correspondence analysis. It identifies the vocabulary statistically overrepresented in maintenance, monitoring, and management across Italian and Spanish interviews, highlighting the lexical contributions driving cross-national differentiation.

|  | **Theme**  **Imaintenance** | **Theme**  **Imanagement** | **Theme**  **Imonitoring** | **Theme**  **Smaintenance** | **Theme**  **Smanagement** | **Theme**  **Smonitoring** |
| --- | --- | --- | --- | --- | --- | --- |
| **live** | -0,8639 | -1,2681 | -0,4591 | 3,2575 | 0,3827 | -0,6392 |
| **osteoporosis** | 1,3716 | 3,2326 | 2,8124 | -4,2955 | -1,1804 | -2,1366 |
| **discomfort** | -0,8424 | 1,1677 | 2,1721 | -3,4736 | -0,2253 | 0,5106 |
| **year** | -1,7827 | -0,3576 | -0,7625 | -1,576 | 1,0164 | 4,9002 |
| **ago** | -0,37 | -0,5446 | -0,8864 | 0,5936 | -0,1806 | 1,1218 |
| **fracture** | -1,0808 | 2,1505 | 1,2986 | -2,1853 | -1,0156 | 0,8974 |
| **move** | 0,7542 | -0,6363 | 0,7156 | -0,6097 | -0,2482 | 0,258 |
| **improve** | -0,4776 | 1,0409 | 0,7818 | -1,6647 | -0,2754 | 0,5882 |
| **blood** | -2,8685 | -0,9598 | 0,7099 | -0,5475 | 2,1734 | 0,9815 |
| **movement** | 1,5085 | 0,2856 | 1,2613 | -0,6334 | -1,0793 | -1,2568 |
| **care** | 0,6695 | -0,6473 | -0,4591 | 1,0492 | -0,2323 | -0,6392 |
| **due** | 1,2694 | 0,3076 | 1,2335 | -1,6396 | -0,4129 | -0,542 |
| **relate** | -0,418 | 1,8083 | 2,2227 | -1,5258 | -0,6591 | -0,7675 |
| **follow** | 3,4063 | -1,2906 | -0,3553 | 2,1528 | -2,9956 | -2,7905 |
| **treatment** | 0,8113 | 3,2094 | -0,5533 | -1,3594 | 0,4233 | -2,2179 |
| **result** | -1,3012 | 1,2172 | 0,2591 | -1,2583 | 0,9324 | 0,4109 |
| **forget** | 0,3703 | -0,9153 | -0,2774 | 1,4139 | -0,7791 | 0,2253 |
| **alendronate** | 4,5861 | -0,3514 | 0,3668 | -1,6647 | -0,7191 | -0,8373 |
| **prescribe** | -0,3249 | 0,3301 | 0,3868 | -1,1724 | 1,0319 | 0,3386 |
| **body** | 0,4962 | 0,2958 | 0,8685 | -0,8144 | -0,2393 | -0,3022 |
| **diet** | 8,7823 | -4,8172 | -3,7889 | 4,2826 | -4,0999 | -4,7745 |
| **medication** | -0,6059 | 2,6408 | 1,996 | -1,2818 | -0,5475 | -1,4057 |
| **engage** | 3,3509 | 0,343 | -0,1806 | -1,387 | -0,5991 | -0,6976 |
| **physical** | 4,4135 | -0,2658 | 0,5333 | -1,5339 | -0,7349 | -1,6069 |
| **activity** | 4,6972 | 0,3745 | -0,3007 | -0,9581 | -1,3197 | -1,5368 |
| **exercise** | 3,0137 | -0,9598 | -1,0901 | 1,051 | -1,2102 | -0,9462 |
| **feel** | -4,797 | 0,6007 | -0,6848 | -1,1408 | 0,3699 | 7,0121 |
| **significant** | 0,249 | 2,0154 | 0,9682 | -1,387 | -0,5991 | -0,6976 |
| **daily** | 1,5582 | -0,187 | 0,2904 | 0,2529 | -0,8391 | -0,9771 |
| **life** | -0,6735 | -0,2382 | 0,3014 | 0,6344 | 0,2579 | -0,2333 |
| **stable** | 0,3531 | -0,2945 | -0,3571 | 0,3045 | -0,2034 | 0,3137 |
| **medical** | 0,2656 | 5,0398 | 0,6247 | -1,9653 | -1,2596 | -1,4668 |
| **advice** | -0,2413 | 3,8132 | -0,2774 | -0,5738 | -0,7791 | -0,9072 |
| **advise** | 4,7843 | 0,2545 | -0,2226 | -1,7255 | -1,1394 | -0,6912 |
| **doctor** | 0,2656 | -0,4289 | -1,1641 | -0,3288 | 0,9092 | 0,7202 |
| **manage** | 0,6217 | 6,0564 | -1,1481 | -2,8572 | 0,4848 | -1,7035 |
| **hospital** | 1,1587 | 1,0905 | 2,0608 | -2,9165 | -1,2596 | -0,7972 |
| **physiotherapist** | 0,249 | -0,7039 | -0,5537 | 1,5196 | 0,4253 | -0,6976 |
| **time** | -0,9745 | -0,3744 | -1,3499 | 1,6858 | 0,297 | 0,5015 |
| **general_practitioner** | -0,236 | 14,8895 | -0,6794 | -4,7293 | -2,0423 | -2,3782 |
| **trust** | -0,4776 | 2,3462 | -0,6645 | -0,2393 | 0,3283 | -0,8373 |
| **therapy** | 4,6535 | 0,7984 | 0,2297 | -2,4542 | -1,5002 | -1,0164 |
| **understand** | -0,2028 | 1,6301 | -0,6645 | -0,4918 | 0,3283 | -0,3468 |
| **week** | 3,0935 | -1,1076 | -1,9989 | 0,5845 | -0,855 | -0,2578 |
| **pill** | -1,2248 | -1,2599 | -2,1661 | 2,9144 | 2,0851 | -0,8265 |
| **remember** | 1,1618 | -0,9153 | -0,72 | 0,5729 | -0,3128 | 0,2253 |
| **drink** | -0,3514 | -1,904 | -1,4977 | 3,9629 | 0,9052 | -1,8871 |
| **stomach** | 0,285 | -0,6363 | -0,2066 | -1,3168 | -1,5604 | 4,6184 |
| **morning** | 0,4295 | -1,4689 | -0,3951 | 2,276 | -0,4755 | -0,6683 |
| **day** | 0,333 | -5,8985 | -2,9016 | 11,0803 | -3,2633 | 0,267 |
| **end** | 0,249 | -0,2627 | -0,5537 | -0,3398 | 0,4253 | 0,7566 |
| **regular** | -0,3602 | 0,7489 | -0,1806 | 0,5524 | -0,5991 | -0,2592 |
| **put** | -0,4776 | -0,3514 | -0,6645 | -0,2393 | 2,6851 | -0,3468 |
| **find** | 2,1377 | -0,4289 | -0,2738 | 0,4655 | -0,645 | -1,4668 |
| **glass** | -0,3602 | -0,7039 | -0,5537 | 4,2029 | -0,5991 | -0,6976 |
| **thing** | -2,2917 | -1,5451 | -1,7193 | 1,136 | 2,3764 | 1,1836 |
| **eat** | 1,0312 | -6,1763 | -3,7418 | 18,7528 | -5,2565 | -4,9051 |
| **breakfast** | 1,049 | -0,7039 | -0,5537 | 0,2654 | -0,2045 | 0,3474 |
| **routine** | 3,2196 | -0,3979 | -0,72 | -0,5738 | 0,2896 | -0,9072 |
| **visit** | -0,6195 | 2,7166 | -0,5769 | -2,9165 | -0,3182 | 1,5713 |
| **hour** | 0,2656 | -0,807 | -0,5769 | 1,4313 | 0,2845 | -0,422 |
| **pain** | -8,1843 | 2,5733 | 3,3468 | -18,9632 | 8,0967 | 1,412 |
| **wait** | -0,4776 | -0,3514 | 0,7818 | 0,373 | -0,2754 | 0,2596 |
| **difficult** | -0,6016 | 0,456 | 2,4352 | -0,6591 | -0,8391 | -0,1837 |
| **stand** | 0,2782 | -0,4947 | 0,5792 | -1,2583 | 0,9324 | 0,4109 |
| **specific** | 7,1006 | -0,9167 | -0,3285 | -1,045 | -0,7349 | -1,6069 |
| **stay** | 2,3114 | -0,9692 | 0,4112 | -0,6001 | -1,1035 | 0,3637 |
| **active** | 8,3102 | -1,0286 | -0,3864 | -0,8351 | -1,5002 | -1,747 |
| **walk** | 2,1343 | -3,5597 | -1,2057 | 3,0084 | -0,8887 | -0,8415 |
| **leg** | -0,3396 | 0,4134 | 1,0144 | -0,5515 | -0,645 | 0,4204 |
| **problem** | -1,7028 | -0,2255 | 0,7144 | -0,6494 | 1,1417 | 0,5942 |
| **tire** | -0,7287 | -0,9167 | -1,2752 | 0,5587 | 2,8545 | -0,2604 |
| **leave** | -0,6195 | -0,807 | 0,6247 | 1,8927 | -0,645 | -0,422 |
| **sit** | -0,6735 | -1,5506 | -0,3007 | 1,2862 | 0,5058 | 0,3813 |
| **moc_scan** | -0,3251 | 1,2172 | 2,2589 | -2,0817 | -0,3908 | -0,4883 |
| **physically** | 5,2209 | -0,9153 | 0,326 | -1,0327 | -0,7791 | -0,9072 |
| **test** | -2,3476 | -0,3881 | 0,4575 | -0,6997 | 0,5657 | 2,5334 |
| **side_effects** | -1,2441 | 3,2669 | 3,4202 | -6,195 | -0,5405 | 0,3894 |
| **experience** | -2,1477 | 8,1796 | 7,922 | -6,195 | -2,2927 | -3,7856 |
| **symptom** | -2,4325 | 0,3872 | 4,4214 | -3,8919 | 0,3055 | 0,9614 |
| **long** | -0,4609 | -0,8171 | 0,3094 | -0,6044 | 1,0878 | 0,8378 |
| **monitor** | -1,3549 | 0,2788 | 5,9244 | -3,4736 | -0,4461 | 0,5106 |
| **start** | -0,3866 | 0,3872 | -0,8696 | 0,6738 | -0,2966 | 0,395 |
| **stop** | -2,9558 | -0,3869 | -1,1351 | 3,4015 | -0,461 | 0,6362 |
| **osteoporosis_specialist** | -3,5829 | -1,6562 | -1,4433 | -0,5602 | 3,9727 | 4,0181 |
| **recently** | 0,249 | 0,7489 | -0,1806 | -0,7087 | -0,2045 | 0,3474 |
| **additional** | 0,5767 | 1,3077 | 0,9682 | -1,387 | -0,5991 | -0,6976 |
| **consultation** | -0,3602 | -0,2627 | -0,1806 | -0,3398 | -0,2045 | 1,3192 |
| **month** | 0,2615 | -0,577 | -1,0411 | 1,0508 | 1,0541 | -0,8107 |
| **appointment** | -0,3602 | 0,343 | 0,4683 | -0,3398 | -0,2045 | 0,3474 |
| **change** | -0,2666 | -0,3531 | -0,2226 | -0,426 | -0,2599 | 1,2942 |
| **give** | -0,785 | -0,9724 | -1,3308 | 1,4029 | 2,0967 | -0,542 |
| **worsen** | -0,5389 | 2,9138 | 1,8663 | -1,8037 | -0,7791 | -0,9072 |
| **condition** | 1,0312 | 4,0093 | 2,2373 | -4,3585 | -1,5614 | -2,7998 |
| **bone** | 0,4993 | 0,8283 | 0,257 | -1,7684 | 0,3605 | 0,3904 |
| **lose** | -0,2327 | -1,2681 | -0,4591 | -0,368 | -0,5146 | 3,3423 |
| **enjoy** | 1,3049 | -0,8448 | -0,6645 | 0,6805 | 0,3283 | -0,8373 |
| **undergo** | 0,4876 | 0,6819 | 2,8356 | -1,3733 | -0,9592 | -1,1169 |
| **receive** | 0,6695 | -0,317 | -0,4591 | 0,4375 | 0,3827 | -0,6392 |
| **explain** | -0,4776 | -0,3514 | -0,2439 | 0,6805 | -0,2754 | 0,5882 |
| **health** | 2,3805 | 0,5964 | -0,3285 | -1,045 | -0,3805 | -0,9057 |
| **maintain** | 5,6139 | 0,2505 | -0,6446 | -0,5475 | -1,2102 | -2,308 |
| **calcium** | -0,2446 | -2,0455 | -0,2701 | 3,4269 | -1,741 | -0,2472 |
| **vitamin_d** | 5,3746 | -1,7336 | 0,8367 | -0,549 | -0,8954 | -2,589 |
| **fall** | -1,8718 | -0,5624 | -0,3403 | 0,279 | -0,4033 | 3,4696 |
| **control** | -1,1275 | -0,3979 | 0,326 | -0,295 | -0,3128 | 2,1531 |
| **denosumab** | -0,5401 | 0,7173 | 0,4754 | 0,4305 | -0,4033 | -0,5525 |
| **avoid** | 2,9455 | -0,7531 | -1,1085 | 1,1683 | -1,1995 | -1,3968 |
| **schedule** | -0,3251 | 0,405 | 1,5861 | -1,2583 | -0,3908 | 0,4109 |
| **check_ups** | -0,9538 | 1,1644 | 4,1408 | -1,5258 | -0,6591 | -0,7675 |
| **recommendation** | 1,3049 | 0,2559 | 0,7818 | -0,9226 | -0,2754 | -0,8373 |
| **wrong** | -1,2143 | -0,187 | 6,4487 | -1,1447 | -0,8391 | -0,44 |
| **notice** | -3,7492 | 0,3341 | 1,5202 | -5,4184 | 1,0098 | 4,6366 |
| **issue** | -0,3249 | 3,5751 | 1,1933 | -1,9478 | -0,7512 | -0,9971 |
| **mention** | -0,6655 | 0,7558 | -0,3475 | -1,2583 | 1,4611 | 0,4109 |
| **case** | -1,3012 | -0,2172 | -0,831 | -0,2159 | 2,8442 | -0,2134 |
| **read** | -0,3602 | -0,2627 | 0,4683 | 0,5524 | -0,5991 | 0,3474 |
| **inform** | -0,867 | 0,7489 | -0,1806 | -0,3398 | 0,8939 | -0,2592 |
| **person** | -0,2853 | -0,593 | 0,2297 | 0,432 | -0,2253 | 0,5106 |
| **friend** | -0,3602 | 0,7489 | -0,5537 | -0,3398 | 0,4253 | 0,3474 |
| **happen** | -0,867 | -0,7039 | -0,5537 | 0,2654 | 0,4253 | 2,0307 |
| **severe** | 0,3208 | 3,5059 | 0,6387 | -1,9427 | -0,8391 | -0,9771 |
| **weight** | 2,5529 | -1,5506 | -1,2197 | 2,2073 | -1,3197 | -1,5368 |
| **gain** | 2,4296 | -0,7039 | -0,5537 | 0,5524 | -0,5991 | -0,6976 |
| **vegetable** | 1,9288 | -0,8448 | -0,6645 | 1,0989 | -0,7191 | -0,8373 |
| **milk** | 0,6708 | -2,6121 | -2,0546 | 8,9502 | -2,2232 | -2,589 |
| **coffee** | 0,4282 | -0,8448 | -0,6645 | 0,373 | 1,8976 | -0,8373 |
| **half** | 0,5767 | -0,7039 | -0,5537 | 0,2654 | -0,5991 | 1,3192 |
| **fruit** | 0,3703 | -0,9153 | -0,72 | 3,5589 | -0,7791 | -0,9072 |
| **age** | 0,6244 | -0,4457 | 2,4352 | -1,1447 | 0,256 | -0,9771 |
| **back** | -1,2033 | -0,792 | 0,3215 | -0,4315 | 2,8666 | 0,2726 |
| **hip** | 1,049 | -0,7039 | 0,4683 | -1,387 | 0,4253 | 0,3474 |
| **special** | -0,9319 | 1,2792 | -0,2226 | -0,426 | 0,6538 | -0,3473 |
| **tablet** | -0,7287 | -1,6212 | -1,2752 | 0,3513 | 1,6639 | 1,3621 |
| **immediately** | -1,0406 | 2,3462 | 0,7818 | -0,9226 | 0,3283 | -0,8373 |
| **normal** | -0,7306 | -0,249 | 1,4671 | -0,2627 | -0,4312 | 0,3658 |
| **work** | -0,2186 | -0,4207 | -0,4787 | 0,2907 | 1,2258 | -0,4129 |
| **remain** | 0,2782 | 1,2172 | 1,0241 | -2,0817 | -0,3908 | -0,2134 |
| **consult** | -1,0703 | 9,2813 | -0,5769 | -2,9165 | -0,3182 | -0,7972 |
| **osteoporosis_specialists** | 0,3929 | 4,503 | 0,8809 | -3,195 | -1,3799 | -0,9057 |
| **recommend** | 0,4962 | 0,2958 | -0,2116 | 0,4535 | -0,6591 | -0,3022 |
| **bad** | -0,37 | -1,1269 | -0,3839 | 0,343 | -0,4312 | 2,2881 |
| **injection** | -0,7117 | -0,2644 | 0,3622 | 0,2688 | 0,2995 | 0,554 |
| **cholesterol** | 0,917 | 0,6586 | -0,6091 | 0,4535 | -0,6591 | -0,7675 |
| **sun** | 1,004 | -1,904 | -1,4977 | 3,9629 | -0,5148 | -1,8871 |
| **exposure** | 3,3509 | -0,7039 | -0,5537 | 0,2654 | -0,5991 | -0,6976 |
| **hurt** | -1,5739 | -1,2 | -1,5533 | -0,492 | 2,1854 | 2,869 |
| **dairy** | 1,6656 | -0,7039 | -0,5537 | 0,9671 | -0,5991 | -0,6976 |
| **product** | 0,8046 | -0,8448 | -0,6645 | 2,2984 | -0,7191 | -0,8373 |
| **home** | -0,4609 | -2,1163 | -1,6646 | 5,8921 | 0,2583 | -1,3011 |
| **rest** | -1,0406 | 2,3462 | -0,2439 | -0,9226 | 1,2389 | -0,8373 |
| **disease** | -1,3012 | -0,4947 | 0,2591 | -0,7475 | 0,5188 | 2,4794 |
| **swim** | 0,8046 | -0,8448 | -0,6645 | 0,6805 | 0,7133 | -0,8373 |
| **return** | -0,867 | -0,2627 | 0,9682 | 0,9671 | -0,5991 | -0,2592 |
| **adverse** | -0,9538 | -0,7743 | 2,2227 | -1,5258 | -0,2393 | 1,8232 |
| **reaction** | -0,418 | -0,3063 | 2,2227 | -1,5258 | -0,6591 | 1,1754 |
| **good** | -1,5921 | -0,7575 | -1,2716 | 3,2972 | 1,3879 | -1,1419 |
| **bite** | -1,0199 | -1,2 | -0,8696 | -0,3116 | 1,6727 | 1,7904 |
| **small** | -0,3602 | -0,7039 | -0,5537 | 0,5524 | 0,8939 | 0,3474 |
| **break** | -0,6735 | -0,8616 | -0,3007 | -0,6186 | 0,8429 | 1,9806 |
| **floor** | -1,0406 | -0,3514 | -0,6645 | 5,2579 | -0,7191 | -0,8373 |
| **general** | -1,4274 | -0,6363 | -0,4165 | -0,9119 | 2,4271 | 1,5263 |
| **worry** | -0,418 | 0,2958 | -0,2116 | -0,4137 | -0,2393 | 1,1754 |
| **effect** | -0,6016 | -0,4457 | 0,2904 | -1,9427 | 1,0227 | 1,9673 |
| **bed** | -1,1275 | -0,9153 | 0,7058 | 1,4139 | 0,2896 | -0,3927 |
| **love** | 0,249 | -0,7039 | -0,5537 | 0,2654 | 2,299 | -0,6976 |
| **cheese** | -0,4193 | -1,6212 | -1,2752 | 8,4565 | -1,3799 | -1,6069 |
| **hard** | -0,9538 | -0,7743 | -0,6091 | -0,1884 | 0,3731 | 2,6079 |
| **lie** | -0,5389 | -0,9153 | -0,72 | 1,4139 | 1,7344 | -0,9072 |
| **implant** | -0,867 | 0,7489 | 0,9682 | 0,2654 | -0,5991 | -0,2592 |
| **meat** | -0,3602 | -0,7039 | -0,5537 | 4,2029 | -0,5991 | -0,6976 |
| **begin** | -0,867 | -0,7039 | -0,5537 | 0,2654 | 0,4253 | 2,0307 |
| **paracetamol** | -1,562 | 0,2856 | -0,4591 | -1,607 | 4,7136 | 0,2906 |
| **call** | -1,0406 | 0,2559 | -0,6645 | -0,4918 | 1,8976 | 0,5882 |
| **fine** | -1,7359 | -0,7531 | -0,537 | -0,4872 | -0,2886 | 5,3832 |
| **height** | -0,6016 | -0,9858 | -0,7755 | -0,6591 | -0,3513 | 5,5601 |
| **afternoon** | -0,867 | -0,7039 | -0,1806 | 2,2234 | -0,2045 | -0,2592 |
| **night** | -1,475 | -1,1975 | -0,942 | 3,6109 | -0,4725 | 0,6245 |
| **truth** | -0,867 | -0,7039 | -0,5537 | -0,3398 | 3,2291 | 0,3474 |
| **kind** | -0,867 | -0,7039 | -0,5537 | 0,9671 | 0,8939 | 0,3474 |
| **fish** | -1,1275 | -0,9153 | -0,72 | 7,3455 | -0,7791 | -0,9072 |
| **moc** | -1,3012 | -1,0564 | -0,831 | 0,407 | 0,5188 | 2,4794 |
| **practitioner** | -1,562 | -1,2681 | -0,9975 | -0,6334 | 3,016 | 1,9604 |
| **daughter** | -1,1275 | -0,9153 | -0,72 | 2,0014 | 0,6401 | 0,2253 |
| **buy** | -0,9538 | -0,7743 | -0,6091 | 6,2128 | -0,6591 | -0,7675 |
| **yogurt** | -1,3012 | -1,0564 | -0,831 | 8,4792 | -0,8991 | -1,047 |
