# Supplementary material for: Self‐Care Behaviors in Osteoporosis: A Cross‐Cultural Study Using Multidimensional Analysis of Textual Data
Source: J Nurs Scholarsh. 2026 Sep 14;58(5):e70134. doi: 10.1111/jnu.70134 (PMC13575457; doi:10.1111/jnu.70134)
Supplement: Supplementary file 3 [file JNU-58-0-s003.docx]

Table S2: Lexical profile for the Italian subsamples

**Supplementary material**

| **Word** | **F** | **Word** | **F** | **Word** | **F** | **Word** | **F** | **Word** | **F** | **Word** | **F** |
| --- | --- | --- | --- | --- | --- | --- | --- | --- | --- | --- | --- |
| *osteoporosis* | 285 | *risk* | 23 | *beneficial* | 14 | *end* | 9 | *bring* | 6 | *begin* | 2 |
| *medication* | 118 | *fall* | 22 | *weight* | 13 | *lose* | 9 | *instruction* | 6 | *hand* | 2 |
| *treatment* | 100 | *week* | 22 | *friend* | 13 | *sun* | 9 | *vegetable* | 6 | *physiotherapist* | 2 |
| *pain* | 94 | *advice* | 22 | *aware* | 13 | *woman* | 9 | *step* | 6 | *house* | 2 |
| *condition* | 91 | *management* | 22 | *cautious* | 13 | *stand* | 9 | *sign* | 6 | *medicine* | 2 |
| *feel* | 88 | *exercise* | 21 | *center* | 13 | *complication* | 9 | *return* | 6 | *relax* | 2 |
| *year* | 76 | *change* | 21 | *wrong* | 13 | *treat* | 9 | *strong* | 6 | *arm* | 2 |
| *manage* | 75 | *discomfort* | 21 | *concern* | 13 | *fear* | 9 | *decide* | 6 | *call* | 2 |
| *care* | 73 | *consult* | 21 | *system* | 13 | *precaution* | 9 | *usual* | 6 | *place* | 2 |
| *experience* | 65 | *visit* | 20 | *challenge* | 13 | *show* | 9 | *gain* | 6 | *small* | 2 |
| *walk* | 56 | *difficult* | 20 | *femur* | 13 | *constantly* | 9 | *bite* | 5 | *calm* | 2 |
| *fracture* | 55 | *body* | 20 | *stop* | 12 | *attend* | 9 | *home* | 5 | *jump* | 2 |
| *follow* | 54 | *diagnosis* | 20 | *blood* | 12 | *exposure* | 9 | *break* | 5 | *prefer* | 2 |
| *bone* | 51 | *remain* | 20 | *negative* | 12 | *develop* | 9 | *happen* | 5 | *simple* | 2 |
| *medical* | 49 | *worsen* | 20 | *high* | 12 | *benefit* | 9 | *general* | 5 | *tooth* | 2 |
| *diet* | 48 | *seek* | 20 | *simply* | 12 | *proper* | 9 | *afraid* | 5 | *gym* | 2 |
| *time* | 47 | *denosumab* | 19 | *clear* | 12 | *hour* | 8 | *child* | 5 | *bicycle* | 2 |
| *Osteoporosis specialist* | 46 | *continue* | 19 | *self_care* | 12 | *worry* | 8 | *effect* | 5 | *floor* | 1 |
| *health* | 46 | *aspect* | 19 | *include* | 12 | *mention* | 8 | *side* | 5 | *lie* | 1 |
| *issue* | 45 | *prevent* | 19 | *ensure* | 12 | *limitation* | 8 | *wake* | 5 | *carry* | 1 |
| *hospital* | 45 | *require* | 19 | *well_being* | 12 | *attention* | 8 | *adverse* | 5 | *afternoon* | 1 |
| *side_effects* | 43 | *injection* | 18 | *give* | 11 | *pay* | 8 | *dairy* | 5 | *god* | 1 |
| *general_practitioner* | 43 | *receive* | 18 | *normal* | 11 | *consultation* | 8 | *drink* | 4 | *suppose* | 1 |
| *maintain* | 41 | *appointment* | 18 | *leave* | 11 | *gymnastics* | 8 | *sit* | 4 | *height* | 1 |
| *activity* | 41 | *lead* | 18 | *explain* | 11 | *low* | 8 | *case* | 4 | *pilates* | 1 |
| *osteoporosis_specialists* | 40 | *move* | 17 | *important* | 11 | *main* | 8 | *put* | 4 | *today* | 1 |
| *vitamin_d* | 39 | *careful* | 17 | *inform* | 11 | *implant* | 8 | *limit* | 4 | *meat* | 1 |
| *prescribe* | 38 | *leg* | 17 | *trust* | 11 | *check_up* | 8 | *knee* | 4 | *terrible* | 1 |
| *check_ups* | 37 | *understand* | 17 | *support* | 11 | *past* | 8 | *paracetamol* | 4 | *glass* | 1 |
| *therapy* | 37 | *early* | 17 | *recently* | 11 | *dentist* | 8 | *control* | 4 | *travel* | 1 |
| *undergo* | 37 | *routine* | 17 | *regular* | 11 | *clinic* | 8 | *foot* | 4 | *turn* | 1 |
| *live* | 36 | *improve* | 17 | *learn* | 11 | *factor* | 8 | *half* | 4 | *sleep* | 1 |
| *stay* | 36 | *physically* | 17 | *spine* | 11 | *yoga* | 8 | *food* | 4 | *depend* | 1 |
| *due* | 36 | *patient* | 17 | *specialist* | 11 | *pill* | 7 | *run* | 4 | *strange* | 1 |
| *physical* | 36 | *alendronate* | 17 | *face* | 11 | *doctor* | 7 | *check* | 4 | *solution* | 1 |
| *moc_scan* | 35 | *back* | 16 | *specialize* | 11 | *swim* | 7 | *climb* | 4 | *grind* | 1 |
| *active* | 33 | *ago* | 16 | *good* | 10 | *remember* | 7 | *refer* | 4 | *truth* | 0 |
| *avoid* | 31 | *morning* | 16 | *stomach* | 10 | *barrier* | 7 | *product* | 4 | *practitioner* | 0 |
| *diagnose* | 31 | *special* | 16 | *enjoy* | 10 | *fact* | 7 | *big* | 4 | *daughter* | 0 |
| *thing* | 29 | *result* | 16 | *hip* | 10 | *read* | 7 | *reach* | 4 | *night* | 0 |
| *problem* | 28 | *daily* | 16 | *wait* | 10 | *rest* | 7 | *breakfast* | 4 | *pool* | 0 |
| *life* | 27 | *schedule* | 16 | *easy* | 10 | *family* | 7 | *honestly* | 4 | *kind* | 0 |
| *start* | 27 | *engage* | 16 | *part* | 10 | *progress* | 7 | *strengthen* | 4 | *moc* | 0 |
| *month* | 27 | *work* | 15 | *mind* | 10 | *great* | 7 | *mother* | 4 | *yogurt* | 0 |
| *advise* | 27 | *bad* | 15 | *situation* | 10 | *sister* | 7 | *hurt* | 3 | *fish* | 0 |
| *specific* | 27 | *stable* | 15 | *menopause* | 10 | *sport* | 7 | *wear* | 3 | *stretch* | 0 |
| *day* | 26 | *recommend* | 15 | *balance* | 10 | *minute* | 7 | *cheese* | 3 | *anymore* | 0 |
| *eat* | 26 | *hope* | 15 | *cholesterol* | 10 | *period* | 7 | *bed* | 3 | *buy* | 0 |
| *long* | 26 | *immediately* | 15 | *professional* | 10 | *dental* | 7 | *moment* | 3 | *osteoarthritis* | 0 |
| *person* | 26 | *recommendation* | 15 | *prevention* | 10 | *play* | 7 | *stair* | 3 | *village* | 0 |
| *find* | 26 | *healthy* | 15 | *osteopenia* | 10 | *reaction* | 7 | *spend* | 3 | *son* | 0 |
| *symptom* | 26 | *approach* | 15 | *difficulty* | 10 | *level* | 7 | *dance* | 3 | *talk* | 0 |
| *monitor* | 26 | *healthcare* | 15 | *fortunately* | 10 | *density* | 7 | *coffee* | 3 | *hit* | 0 |
| *movement* | 26 | *plan* | 15 | *rely* | 10 | *fine* | 6 | *fruit* | 3 | *inject* | 0 |
| *lifestyle* | 26 | *additional* | 15 | *initially* | 10 | *tire* | 6 | *bother* | 3 | *shoe* | 0 |
| *calcium* | 25 | *disease* | 14 | *significantly* | 10 | *affect* | 6 | *pass* | 3 |  |  |
| *severe* | 25 | *positive* | 14 | *adjust* | 10 | *hard* | 6 | *point* | 3 |  |  |
| *test* | 24 | *suffer* | 14 | *reassure* | 10 | *forget* | 6 | *love* | 3 |  |  |
| *notice* | 24 | *moc_scans* | 14 | *t_score* | 10 | *husband* | 6 | *surgery* | 3 |  |  |
| *significant* | 24 | *relate* | 14 | *milk* | 9 | *young* | 6 | *tablet* | 2 |  |  |

Note: F stands for Frequency
