# Supplementary material for: Self‐Care Behaviors in Osteoporosis: A Cross‐Cultural Study Using Multidimensional Analysis of Textual Data
Source: J Nurs Scholarsh. 2026 Sep 14;58(5):e70134. doi: 10.1111/jnu.70134 (PMC13575457; doi:10.1111/jnu.70134)
Supplement: Supplementary file 4 [file JNU-58-0-s004.docx]

**Supplementary material**

Table S1: Structure and meaning of metadata lines used for corpus segmentation and lexicometric analysis.

Each metadata line precedes a textual segment and provides structured information to the software for statistical processing. Metadata are introduced by an asterisk (*) and function as categorical variables that allow the association of text segments with participant characteristics and theoretical domains, without manual coding. Specifically, speaker_I01 identifies the individual participant (I = Italian participant; S = Spanish participant; numeric code = participant ID). sex_F or sex_M indicates the participant’s biological sex (female or male). age_68 refers to the participant’s age in years at the time of the interview. theme_Imaintenance, theme_Imonitoring, or theme_Imanagement specify the self-care domain addressed in that portion of the interview, in accordance with the Middle-Range Theory of Self-Care of Chronic Illness; the prefix I or S denotes whether the segment refers to an Italian or Spanish participant. Finally, naz_ita or naz_esp indicates the participant’s nationality (Italian or Spanish). Each interview was segmented into three distinct textual units corresponding to self-care maintenance, monitoring, and management, allowing the software to analyse how different participant characteristics and theoretical domains were associated with specific lexical patterns.

| **METADATALINE** | |
| --- | --- |
| *speaker_I01 *sex_F *age_68 *theme_Imaintenance *naz_ita  *speaker_I01 *sex_F *age_68 *theme_Imonitoring *naz_ita  *speaker_I01 *sex_F *age_68 *theme_Imanagement *naz_ita | *speaker_S01 *sex_F *age_60 *theme_Smaintenance *naz_esp  *speaker_S01 *sex_F *age_60 *theme_Smonitoring *naz_esp  *speaker_S01 *sex_F *age_60 *theme_Smanagement *naz_esp |
| *speaker_I02 *sex_M *age_64 *theme_Imaintenance *naz_ita  *speaker_I02 *sex_M *age_64 *theme_Imonitoring *naz_ita  *speaker_I02 *sex_M *age_64 *theme_Imanagement *naz_ita | *speaker_S02 *sex_F *age_82 *theme_Smaintenance *naz_esp  *speaker_S02 *sex_F *age_82 *theme_Smonitoring *naz_esp  *speaker_S02 *sex_F *age_82 *theme_Smanagement *naz_esp |
| *speaker_I03 *sex_F *age_67 *theme_Imaintenance *naz_ita  *speaker_I03 *sex_F *age_67 *theme_Imonitoring *naz_ita  *speaker_I03 *sex_F *age_67 *theme_Imanagement *naz_ita | *speaker_S03 *sex_F *age_70 *theme_Smaintenance *naz_esp  *speaker_S03 *sex_F *age_70 *theme_Smonitoring *naz_esp  *speaker_S03 *sex_F *age_70 *theme_Smanagement *naz_esp |
| *speaker_I04 *sex_F *age_59 *theme_Imaintenance *naz_ita  *speaker_I04 *sex_F *age_59 *theme_Imonitoring *naz_ita  *speaker_I04 *sex_F *age_59 *theme_Imanagement *naz_ita | *speaker_S04 *sex_F *age_65 *theme_Smaintenance *naz_esp  *speaker_S04 *sex_F *age_65 *theme_Smonitoring *naz_esp  *speaker_S04 *sex_F *age_65 *theme_Smanagement *naz_esp |
| *speaker_I05 *sex_F *age_60 *theme_Imaintenance *naz_ita  *speaker_I05 *sex_F *age_60 *theme_Imonitoring *naz_ita  *speaker_I05 *sex_F *age_60 *theme_Imanagement *naz_ita | *speaker_S05 *sex_F *age_69 *theme_Smaintenance *naz_esp  *speaker_S05 *sex_F *age_69 *theme_Smonitoring *naz_esp  *speaker_S05 *sex_F *age_69 *theme_Smanagement *naz_esp |
| *speaker_I06 *sex_F *age_69 *theme_Imaintenance *naz_ita  *speaker_I06 *sex_F *age_69 *theme_Imonitoring *naz_ita  *speaker_I06 *sex_F *age_69 *theme_Imanagement *naz_ita | *speaker_S06 *sex_F *age_77 *theme_Smaintenance *naz_esp  *speaker_S06 *sex_F *age_77 *theme_Smonitoring *naz_esp  *speaker_S06 *sex_F *age_77 *theme_Smanagement *naz_esp |
| *speaker_I07 *sex_F *age_65 *theme_Imaintenance *naz_ita  *speaker_I07 *sex_F *age_65 *theme_Imonitoring *naz_ita  *speaker_I07 *sex_F *age_65 *theme_Imanagement *naz_ita | *speaker_S07 *sex_F *age_61 *theme_Smaintenance *naz_esp  *speaker_S07 *sex_F *age_61 *theme_Smonitoring *naz_esp  *speaker_S07 *sex_F *age_61 *theme_Smanagement *naz_esp |
| *speaker_I08 *sex_F *age_70 *theme_Imaintenance *naz_ita  *speaker_I08 *sex_F *age_70 *theme_Imonitoring *naz_ita  *speaker_I08 *sex_F *age_70 *theme_Imanagement *naz_ita | *speaker_S08 *sex_F *age_56 *theme_Smaintenance *naz_esp  *speaker_S08 *sex_F *age_56 *theme_Smonitoring *naz_esp  *speaker_S08 *sex_F *age_56 *theme_Smanagement *naz_esp |
| *speaker_I09 *sex_F *age_71 *theme_Imaintenance *naz_ita  *speaker_I09 *sex_F *age_71 *theme_Imonitoring *naz_ita  *speaker_I09 *sex_F *age_71 *theme_Imanagement *naz_ita | *speaker_S09 *sex_F *age_73 *theme_Smaintenance *naz_esp  *speaker_S09 *sex_F *age_73 *theme_Smonitoring *naz_esp  *speaker_S09 *sex_F *age_73 *theme_Smanagement *naz_esp |
| *speaker_I10 *sex_F *age_55 *theme_Imaintenance *naz_ita  *speaker_I10 *sex_F *age_55 *theme_Imonitoring *naz_ita  *speaker_I10 *sex_F *age_55 *theme_Imanagement *naz_ita | *speaker_S10 *sex_F *age_66 *theme_Smaintenance *naz_esp  *speaker_S10 *sex_F *age_66 *theme_Smonitoring *naz_esp  *speaker_S10 *sex_F *age_66 *theme_Smanagement *naz_esp |
| *speaker_I11 *sex_F *age_72 *theme_Imaintenance *naz_ita  *speaker_I11 *sex_F *age_72 *theme_Imonitoring *naz_ita  *speaker_I11 *sex_F *age_72 *theme_Imanagement *naz_ita | *speaker_S11 *sex_F *age_73 *theme_Smaintenance *naz_esp  *speaker_S11 *sex_F *age_73 *theme_Smonitoring *naz_esp  *speaker_S11 *sex_F *age_73 *theme_Smanagement *naz_esp |
| *speaker_I12 *sex_F *age_76 *theme_Imaintenance *naz_ita  *speaker_I12 *sex_F *age_76 *theme_Imonitoring *naz_ita  *speaker_I12 *sex_F *age_76 *theme_Imanagement *naz_ita | *speaker_S12 *sex_F *age_81 *theme_Smaintenance *naz_esp  *speaker_S12 *sex_F *age_81 *theme_Smonitoring *naz_esp  *speaker_S12 *sex_F *age_81 *theme_Smanagement *naz_esp |
| *speaker_I13 *sex_F *age_70 *theme_Imaintenance *naz_ita  *speaker_I13 *sex_F *age_70 *theme_Imonitoring *naz_ita  *speaker_I13 *sex_F *age_70 *theme_Imanagement *naz_ita | *speaker_S13 *sex_F *age_82 *theme_Smaintenance *naz_esp  *speaker_S13 *sex_F *age_82 *theme_Smonitoring *naz_esp  *speaker_S13 *sex_F *age_82 *theme_Smanagement *naz_esp |
| *speaker_I14 *sex_F *age_62 *theme_Imaintenance *naz_ita  *speaker_I14 *sex_F *age_62 *theme_Imonitoring *naz_ita  *speaker_I14 *sex_F *age_62 *theme_Imanagement *naz_ita | *speaker_S14 *sex_F *age_68 *theme_Smaintenance *naz_esp  *speaker_S14 *sex_F *age_68 *theme_Smonitoring *naz_esp  *speaker_S14 *sex_F *age_68 *theme_Smanagement *naz_esp |
| *speaker_I15 *sex_F *age_64 *theme_Imaintenance *naz_ita  *speaker_I15 *sex_F *age_64 *theme_Imonitoring *naz_ita  *speaker_I15 *sex_F *age_64 *theme_Imanagement *naz_ita | *speaker_S15 *sex_M *age_62 *theme_Smaintenance *naz_esp  *speaker_S15 *sex_M *age_62 *theme_Smonitoring *naz_esp  *speaker_S15 *sex_M *age_62 *theme_Smanagement *naz_esp |
| *speaker_I16 *sex_F *age_63 *theme_Imaintenance *naz_ita  *speaker_I16 *sex_F *age_63 *theme_Imonitoring *naz_ita  *speaker_I16 *sex_F *age_63 *theme_Imanagement *naz_ita | *speaker_S16 *sex_F *age_64 *theme_Smaintenance *naz_esp  *speaker_S16 *sex_F *age_64 *theme_Smonitoring *naz_esp  *speaker_S16. *sex_F *age_64 *theme_Smanagement *naz_esp |
| *speaker_I17 *sex_F *age_60 *theme_Imaintenance *naz_ita  *speaker_I17 *sex_F *age_60 *theme_Imonitoring *naz_ita  *speaker_I17 *sex_F *age_60 *theme_Imanagement *naz_ita | *speaker_S17 *sex_F *age_66 *theme_Smaintenance *naz_esp  *speaker_S17 *sex_F *age_66 *theme_Smonitoring *naz_esp  *speaker_S17 *sex_F *age_66 *theme_Smanagement *naz_esp |
| *speaker_I18 *sex_F *age_78 *theme_Imaintenance *naz_ita  *speaker_I18 *sex_F *age_78 *theme_Imonitoring *naz_ita  *speaker_I18 *sex_F *age_78 *theme_Imanagement *naz_ita | *speaker_S18 *sex_F *age_72 *theme_Smaintenance *naz_esp  *speaker_S18 *sex_F *age_72 *theme_Smonitoring *naz_esp  *speaker_S18 *sex_F *age_72 *theme_Smanagement *naz_esp |
| *speaker_I19 *sex_F *age_73 *theme_Imaintenance *naz_ita  *speaker_I19 *sex_F *age_73 *theme_Imonitoring *naz_ita  *speaker_I19 *sex_F *age_73 *theme_Imanagement *naz_ita | *speaker_S19 *sex_M *age_63 *theme_Smaintenance *naz_esp  *speaker_S19 *sex_M *age_63 *theme_Smonitoring *naz_esp  *speaker_S19 *sex_M *age_63 *theme_Smanagement *naz_esp |
| *speaker_I20 *sex_F *age_69 *theme_Imaintenance *naz_ita  *speaker_I20 *sex_F *age_69 *theme_Imonitoring *naz_ita  *speaker_I20 *sex_F *age_69 *theme_Imanagement *naz_ita | *speaker_S20 *sex_F *age_67 *theme_Smaintenance *naz_esp  *speaker_S20 *sex_F *age_67 *theme_Smonitoring *naz_esp  *speaker_S20 *sex_F *age_67 *theme_Smanagement *naz_esp |
